# Supplementary material for: A scoping review of interventions to prevent and treat adverse events during treatment of rifampin-susceptible tuberculosis
Source: PLoS One. 2025 Dec 26;20(12):e0339354. doi: 10.1371/journal.pone.0339354 (PMC12742745; doi:10.1371/journal.pone.0339354)
Supplement: S2 Text — (DOCX) [file pone.0339354.s002.docx]

S2 Text. Details of the PubMed Search:

(((("Tuberculosis"[Mesh] OR "Tuberculoses" OR "Mycobacterium tuberculosis Infection" OR "Infection, Mycobacterium tuberculosis" OR "Infections, Mycobacterium tuberculosis" OR "Mycobacterium tuberculosis Infections" OR "Kochs Disease" OR "Kochs Disease" OR "Koch Disease")) AND (("Antitubercular Agents"[Mesh] OR "Antitubercular Agent" OR "Agent, Antitubercular" OR "Anti-Tuberculosis Agent" OR "Agent, Anti-Tuberculosis" OR "Anti Tuberculosis Agent" OR "Anti-Tuberculosis Drug" OR "Anti Tuberculosis Drug" OR "Drug, Anti-Tuberculosis" OR "Antitubercular Drug" OR "Drug, Antitubercular" OR "Tuberculostatic Agent" OR "Agent, Tuberculostatic" OR "Anti-Tuberculosis Drugs" OR "Anti Tuberculosis Drugs" OR "Anti-Tuberculosis Agents" OR "Anti Tuberculosis Agents" OR "Antitubercular Drugs" OR "Tuberculostatic Agents") OR ("Antitubercular Agents [Pharmacological Action]" OR "essential 303 forte"[tw] OR "thiobenzamide"[tw] OR "sodium thiosulfate"[tw] OR "aconiazide"[tw] OR "thymopoietin III"[tw] OR "sparfloxacin"[tw] OR "pazufloxacin"[tw] OR "isoniazid, pyrazinamide, rifampin drug combination"[tw] OR "thiocarlide"[tw] OR "bedaquiline"[tw] OR "ohmyungsamycin A"[tw] OR "macozinone"[tw] OR "ecumicin"[tw] OR "atratumycin"[tw] OR "perchlozone"[tw] OR "dextrazide"[tw] OR "Ethambutol"[tw] OR "Ethionamide"[tw] OR "Isoniazid"[tw] OR "Aminosalicylic Acid"[tw] OR "Prothionamide"[tw] OR "Pyrazinamide"[tw] OR "Thioacetazone"[tw] OR "Diarylquinolines"[tw] OR "rifapentine"[tw] OR "amprenavir"[tw] OR "KRM 1648"[tw] OR "Mycophenolic Acid"[tw] OR "Rifabutin"[tw] OR "Cycloserine"[tw] OR "Capreomycin"[tw] OR "Rifampin"[tw] OR "Enviomycin"[tw] OR "Viomycin"[tw]))) AND (("Drug-Related Side Effects and Adverse Reactions"[Mesh] OR "Drug Related Side Effects and Adverse Reactions" OR "Side Effects of Drugs" OR "Drug-Related Side Effects and Adverse Reaction" OR "Drug Related Side Effects and Adverse Reaction" OR "Adverse Drug Reaction" OR "Adverse Drug Reactions" OR "Drug Reaction, Adverse" OR "Drug Reactions, Adverse" OR "Reactions, Adverse Drug" OR "Adverse Drug Event" OR "Adverse Drug Events" OR "Drug Event, Adverse" OR "Drug Events, Adverse" OR "Drug Side Effects" OR "Drug Side Effect" OR "Effects, Drug Side" OR "Side Effect, Drug" OR "Side Effects, Drug" OR "Drug Toxicity" OR "Toxicity, Drug" OR "Drug Toxicities" OR "Toxicities, Drug" OR "Nausea"[Mesh] OR Nausea OR "Vomiting"[Mesh] OR Vomiting OR Emesis OR "Chemical and Drug Induced Liver Injury"[Mesh] OR "Hepatitis, Toxic" OR "Toxic Hepatitis" OR "Hepatitides, Toxic" OR "Toxic Hepatitides" OR "Hepatitis, Drug-Induced" OR "Drug-Induced Hepatitides" OR "Drug-Induced Hepatitis" OR "Hepatitides, Drug-Induced" OR "Hepatitis, Drug Induced" OR "Drug-Induced Acute Liver Injury" OR "Drug Induced Acute Liver Injury" OR "Acute Liver Injury, Drug-Induced" OR "Acute Liver Injury, Drug Induced" OR "Liver Injury, Drug-Induced, Acute" OR "Drug-Induced Liver Disease" OR "Disease, Drug-Induced Liver" OR "Diseases, Drug-Induced Liver" OR "Drug Induced Liver Disease" OR "Drug-Induced Liver Diseases" OR "Liver Disease, Drug-Induced" OR "Liver Diseases, Drug-Induced" OR "Drug-Induced Liver Injury" OR "Drug-Induced Liver Injuries" OR "Drug Induced Liver Injury" OR "Injuries, Drug-Induced Liver" OR "Injury, Drug-Induced Liver" OR "Liver Injuries, Drug-Induced" OR "Liver Injury, Drug-Induced" OR "Liver Injury, Drug Induced" OR "Chemically-Induced Liver Toxicity" OR "Chemically-Induced Liver Toxicities" OR "Chemically Induced Liver Toxicity" OR "Liver Toxicities, Chemically-Induced" OR "Liver Toxicity, Chemically-Induced" OR "Toxicities, Chemically-Induced Liver" OR "Toxicity, Chemically-Induced Liver" OR hepatotoxicity OR "Urticaria"[Mesh] OR "Urticarias" OR "Hives" OR "Urticarial Wheals" OR "Urticarial Wheal" OR "Wheals, Urticarial" OR "Wheal, Urticarial" OR photosensitivity OR "Anaphylaxis"[Mesh] OR "Anaphylactic Reaction" OR "Anaphylactic Reactions" OR "Reaction, Anaphylactic" OR "Anaphylactoid Shock" OR "Shock, Anaphylactoid" OR "Anaphylactoid Reaction" OR "Anaphylactoid Reactions" OR "Reaction, Anaphylactoid" OR "Shock, Anaphylactic" OR "Anaphylactic Shock" OR "flu-like syndrome" OR "Toxic Optic Neuropathy"[Mesh] OR "Neuropathy, Toxic Optic" OR "Optic Neuropathy, Toxic" OR "Toxic Optic Neuropathies" OR "Ocular Toxicity" OR "Toxicity, Ocular" OR "Optic Neuritis"[Mesh] OR "Neuritides, Optic" OR "Neuritis, Optic" OR "Optic Neuritides" OR "Retrobulbar Neuritis" OR "Neuritides, Retrobulbar" OR "Neuritis, Retrobulbar" OR "Retrobulbar Neuritides" OR "Posterior Optic Neuritis" OR "Neuritides, Posterior Optic" OR "Neuritis, Posterior Optic" OR "Optic Neuritides, Posterior" OR "Optic Neuritis, Posterior" OR "Posterior Optic Neuritides" OR "Neuropapillitis" OR "Neuropapillitides" OR "Anterior Optic Neuritis" OR "Anterior Optic Neuritides" OR "Neuritides, Anterior Optic" OR "Neuritis, Anterior Optic" OR "Optic Neuritides, Anterior" OR "Optic Neuritis, Anterior" OR "retinal toxicity" OR "Neurotoxicity Syndromes"[Mesh] OR "Neurotoxicity Syndrome" OR "Syndrome, Neurotoxicity" OR "Syndromes, Neurotoxicity" OR "Poisoning, Nervous System" OR "Nervous System Poisonings" OR "Poisonings, Nervous System" OR "Nervous System Poisoning" OR "Neurotoxic Disorders" OR "Neurotoxic Disorder" OR "Neurotoxin Diseases" OR "Neurotoxin Disease" OR "Neurotoxin Disorders" OR "Neurotoxin Disorder" OR "Toxic Encephalitis" OR "Encephalitides, Toxic" OR "Encephalitis, Toxic" OR "Toxic Encephalitides" OR "Encephalopathy, Toxic" OR "Encephalopathies, Toxic" OR "Toxic Encephalopathies" OR "Toxic Encephalopathy" OR "Peripheral Nervous System Diseases"[Mesh] OR "Peripheral Nervous System Disorders" OR "Peripheral Neuropathies" OR "Neuropathy, Peripheral" OR "Peripheral Neuropathy" OR "Peripheral Nervous System Disease" OR "PNS Diseases" OR "PNS Disease" OR "Peripheral Nerve Diseases" OR "Nerve Disease, Peripheral" OR "Nerve Diseases, Peripheral" OR "Peripheral Nerve Disease" OR "PNS Peripheral Nervous System Diseases" OR "Seizures"[Mesh] OR "Seizure" OR "Jacksonian Seizure" OR "Seizure, Jacksonian" OR "Single Seizure" OR "Seizure, Single" OR "Single Seizures" OR "Atonic Absence Seizures" OR "Atonic Absence Seizure" OR "Absence Seizure, Atonic" OR "Absence Seizures, Atonic" OR "Seizure, Atonic Absence" OR "Seizures, Focal" OR "Focal Seizure" OR "Focal Seizures" OR "Seizure, Focal" OR "Partial Seizures" OR "Partial Seizure" OR "Seizure, Partial" OR "Seizures, Generalized" OR "Generalized Seizure" OR "Generalized Seizures" OR "Seizure, Generalized" OR "Seizures, Sensory" OR "Seizure, Sensory" OR "Sensory Seizure" OR "Sensory Seizures" OR "Seizures, Auditory" OR "Auditory Seizure" OR "Auditory Seizures" OR "Seizure, Auditory" OR "Convulsive Seizures" OR "Seizure, Convulsive" OR "Seizures, Convulsive" OR "Seizures, Motor" OR "Motor Seizure" OR "Motor Seizures" OR "Seizure, Motor" OR "Convulsive Seizure" OR "Seizures, Gustatory" OR "Gustatory Seizure" OR "Gustatory Seizures" OR "Seizure, Gustatory" OR "Seizures, Olfactory" OR "Olfactory Seizure" OR "Olfactory Seizures" OR "Seizure, Olfactory" OR "Seizures, Somatosensory" OR "Seizure, Somatosensory" OR "Somatosensory Seizure" OR "Somatosensory Seizures" OR "Seizures, Vertiginous" OR "Seizure, Vertiginous" OR "Vertiginous Seizure" OR "Vertiginous Seizures" OR "Seizures, Vestibular" OR "Seizure, Vestibular" OR "Vestibular Seizure" OR "Vestibular Seizures" OR "Seizures, Visual" OR "Seizure, Visual" OR "Visual Seizure" OR "Visual Seizures" OR "Convulsion, Non-Epileptic" OR "Convulsion, Non Epileptic" OR "Convulsions, Non-Epileptic" OR "Non-Epileptic Convulsion" OR "Non-Epileptic Convulsions" OR "Nonepileptic Seizures" OR "Non-Epileptic Seizures" OR "Non Epileptic Seizures" OR "Nonepileptic Seizure" OR "Seizure, Nonepileptic" OR "Seizures, Nonepileptic" OR "Non-Epileptic Seizure" OR "Non Epileptic Seizure" OR "Seizure, Non-Epileptic" OR "Complex Partial Seizures" OR "Complex Partial Seizure" OR "Partial Seizure, Complex" OR "Partial Seizures, Complex" OR "Seizure, Complex Partial" OR "Epileptic Seizures" OR "Seizures, Epileptic" OR "Epileptic Seizure" OR "Seizure, Epileptic" OR "Generalized Absence Seizures" OR "Generalized Absence Seizure" OR "Absence Seizure, Generalized" OR "Absence Seizures, Generalized" OR "Seizure, Generalized Absence" OR "Tonic-Clonic Seizures" OR "Seizures, Tonic-Clonic" OR "Generalized Tonic-Clonic Seizures" OR "Generalized Tonic-Clonic Seizure" OR "Generalized Tonic Clonic Seizures" OR "Seizure, Generalized Tonic-Clonic" OR "Seizures, Generalized Tonic-Clonic" OR "Tonic-Clonic Seizure, Generalized" OR "Tonic-Clonic Seizures, Generalized" OR "Tonic Clonic Seizure" OR "Clonic Seizures, Tonic" OR "Clonic Seizure, Tonic" OR "Seizure, Tonic Clonic" OR "Tonic Clonic Seizures" OR "Tonic-Clonic Seizure" OR "Seizure, Tonic-Clonic" OR "Myoclonic Seizures" OR "Myoclonic Seizure" OR "Seizure, Myoclonic" OR "Clonic Seizures" OR "Seizures, Clonic" OR "Clonic Seizure" OR "Seizure, Clonic" OR "Tonic Seizures" OR "Seizures, Tonic" OR "Tonic Seizure" OR "Seizure, Tonic" OR "Atonic Seizures" OR "Atonic Seizure" OR "Seizure, Atonic" OR "Convulsions" OR "Convulsion" OR "Absence Seizures" OR "Petit Mal Convulsion" OR "Convulsion, Petit Mal" OR "Absence Seizure" OR "Seizure, Absence" OR "Depression"[Mesh] OR "Depressive Symptoms" OR "Depressive Symptom" OR "Symptom, Depressive" OR "Emotional Depression" OR "Depression, Emotional" OR "Psychotic Disorders"[Mesh] OR "Disorder, Psychotic" OR "Disorders, Psychotic" OR "Psychotic Disorder" OR "Psychoses" OR "Psychosis" OR "Psychosis, Brief Reactive" OR "Brief Reactive Psychoses" OR "Brief Reactive Psychosis" OR "Psychoses, Brief Reactive" OR "Reactive Psychoses, Brief" OR "Reactive Psychosis, Brief" OR "Schizoaffective Disorder" OR "Disorder, Schizoaffective" OR "Disorders, Schizoaffective" OR "Schizoaffective Disorders" OR "Schizophreniform Disorders" OR "Disorder, Schizophreniform" OR "Disorders, Schizophreniform" OR "Schizophreniform Disorder" OR "Ototoxicity"[Mesh] OR "Ototoxicities" OR "Otological Toxicity" OR "Toxicity, Otological" OR "Drug-Induced Ototoxicity" OR "Drug-Induced Ototoxicities" OR "Drug Induced Ototoxicity" OR "Ototoxicities, Drug-Induced" OR "Ototoxicity, Drug-Induced" OR "Drug-Induced Otological Toxicity" OR "Drug-Induced Otological Toxicities" OR "Drug Induced Otological Toxicity" OR "Otological Toxicities, Drug-Induced" OR "Otological Toxicity, Drug-Induced" OR "Drug-Related Otological Toxicity" OR "Drug-Related Otological Toxicities" OR "Drug Related Otological Toxicity" OR "Otological Toxicities, Drug-Related" OR "Otological Toxicity, Drug-Related" OR "Drug-Related Ototoxicity" OR "Drug-Related Ototoxicities" OR "Drug Related Ototoxicity" OR "Ototoxicities, Drug-Related" OR "Ototoxicity, Drug-Related" OR "Drug-Induced Cochleotoxicity" OR "Cochleotoxicities, Drug-Induced" OR "Cochleotoxicity, Drug-Induced" OR "Drug-Induced Cochleotoxicities" OR "Drug Induced Cochleotoxicity" OR "Drug-Induced Cochlear Toxicity" OR "Cochlear Toxicities, Drug-Induced" OR "Cochlear Toxicity, Drug-Induced" OR "Drug-Induced Cochlear Toxicities" OR "Drug Induced Cochlear Toxicity" OR "Drug-Related Cochlear Toxicity" OR "Cochlear Toxicities, Drug-Related" OR "Cochlear Toxicity, Drug-Related" OR "Drug-Related Cochlear Toxicities" OR "Drug Related Cochlear Toxicity" OR "Drug-Related Cochleotoxicity" OR "Cochleotoxicities, Drug-Related" OR "Cochleotoxicity, Drug-Related" OR "Drug-Related Cochleotoxicities" OR "Drug Related Cochleotoxicity" OR "Drug-Induced Vestibulotoxicity" OR "Drug-Induced Vestibulotoxicities" OR "Drug Induced Vestibulotoxicity" OR "Vestibulotoxicities, Drug-Induced" OR "Vestibulotoxicity, Drug-Induced" OR "Drug-Induced Vestibular Toxicity" OR "Drug-Induced Vestibular Toxicities" OR "Drug Induced Vestibular Toxicity" OR "Vestibular Toxicities, Drug-Induced" OR "Vestibular Toxicity, Drug-Induced" OR "Auditory Toxicity" OR "Radiation-Induced Ototoxicity" OR "Ototoxicities, Radiation-Induced" OR "Ototoxicity, Radiation-Induced" OR "Radiation-Induced Ototoxicities" OR "Radiation Induced Ototoxicity" OR "Cytopenia"[Mesh] OR Cytopenia OR Cytopenias))) AND ((clinicaltrial[Filter] OR meta-analysis[Filter] OR randomizedcontrolledtrial[Filter] OR review[Filter] OR "Cohort Studies"[Mesh] OR "Cohort Study" OR "Studies, Cohort" OR "Study, Cohort" OR "Studies, Concurrent" OR "Concurrent Study" OR "Study, Concurrent" OR "Concurrent Studies" OR "Closed Cohort Studies" OR "Cohort Studies, Closed" OR "Closed Cohort Study" OR "Cohort Study, Closed" OR "Study, Closed Cohort" OR "Studies, Closed Cohort" OR "Historical Cohort Studies" OR "Cohort Studies, Historical" OR "Cohort Study, Historical" OR "Historical Cohort Study" OR "Study, Historical Cohort" OR "Studies, Historical Cohort" OR "Incidence Studies" OR "Incidence Study" OR "Studies, Incidence" OR "Study, Incidence" OR "Analysis, Cohort" OR "Analyses, Cohort" OR "Cohort Analyses" OR "Cohort Analysis" OR "Birth Cohort Studies" OR "Birth Cohort Study" OR "Cohort Studies, Birth" OR "Cohort Study, Birth" OR "Studies, Birth Cohort" OR "Study, Birth Cohort"))
