# Supplementary material for: A scoping review of interventions to prevent and treat adverse events during treatment of rifampin-susceptible tuberculosis
Source: PLoS One. 2025 Dec 26;20(12):e0339354. doi: 10.1371/journal.pone.0339354 (PMC12742745; doi:10.1371/journal.pone.0339354)
Supplement: S3 Table — (DOCX) [file pone.0339354.s003.docx]

S3 Table. Characteristics of papers that could not be retrieved

| First author | Journal | Title | Summary of the abstract | Study type |
| --- | --- | --- | --- | --- |
| Abdullaev | Probl Tuberk Bolezn Legk 2009;(2):57-61 | Evaluation of hepatic function in new cases of pulmonary tuberculosis due to the use of standard chemotherapy regimens I and IIB | - Sample size – 147 - Impact of adding kanamycin to INH, rifampin, PZA, and streptomycin or ethambutol - Impact of adding hepatoprotectants - Conclusion – “GGTP is of great informative value in controlling the hepatotoxic effects of control therapy.” | Prevention of hepatoxicity |
| Dai, C. | China Tropical Medicine 2023;23(7):742-747 | Effect of thymopentin combined with levofloxacin for retreatment smear positive pulmonary tuberculosis | - Sample size – 100 - Impact of adding levofloxacin and thymopentin to retreatment regimens - Conclusion – “Thymopentin combined with levofloxacin had a significant application effect in the treatment of retreatment smear positive pulmonary tuberculosis, s, which led to improved inflammatory reaction, respiratory function and immune function.” | Prevention by immunomodulatory therapy |
| Durigato S | Minerva Med Dec 1972;63(91):5024-9 | S-adenosylmethionine in the therapy of liver insufficiency during a course of pulmonary tuberculosis | Not available | Treatment of hepatotoxicity |
| Hu, X | Chinese Traditional and Herbal Drugs 2023;54(13):4245-4252 | Clinical observation of Bufei Huoxue Capsule combined with chemotherapy in treatment of rifampicin-susceptible retreated pulmonary tuberculosis (yin and yang deficiency type) and analysis of prognostic factors | - Sample size – 152 - Impact of  Bufei Huoxue Capsule on re-treatment outcomes - Conclusion – “The use of Bufei Huoxu Capsule in the adjuvant treatment of rifampicin-susceptible retreated patients with pulmonary tuberculosis (yin and yang deficiency type) can improve the success rate of treatment, accelerate lesion absorption, promote symptom improvement, improve cellular immunity, reduce the incidence of adverse reactions…” | Prevention by immunomodulatory therapy |
| Huang, AJ. | Zhonghua Liu Xing Bing Xue Za Zhi Jul 2010;31(7):826-7 | Effectiveness and safety of preventive usage of liver protective drugs during anti-tuberculosis therapy: a systematic review of clinical trials in China | Not available | Prevention of hepatotoxicity |
| Li, J. | Chinese journal of hepatology 2010;18(5):385-386 | Protective effect of silibinin on liver injury induced by antituberculosis drugs | Not available | Prevention of hepatotoxicity |
| Mezhebovskiĭ VR | Probl Tuberk 1990;(10):32-5 | Effectiveness of the treatment of patients with pulmonary tuberculosis using a liver protector zixoryn | - N = 77 - Impact of zixoryn in preventing drug-induced liver injury - Conclusion – “…the liver protector is highly effective in patients with tuberculosis.” | Prevention of hepatotoxicity |
| Mordyk AV | Klin Med (Mosk) 2009;87(5):59-62 | Prevention of neuro- and cardiotoxic side effects of tuberculosis chemotherapy with noopept | - N = 60 - Impact of noopept on adverse events - “The treatment promoted functional normalization of vegetative nervous system and antioxidative systems, reduced manifestations of anxiety, decreased frequency of adverse neuro- and cardiotoxic responses to antituberculous drugs.” | Other form of adverse event prevention |
| Mukhtarov DZ | Probl Tuberk 1998;(5):23-4 | Clinical picture, course and therapeutic efficiency of pulmonary tuberculosis in patients with organochlorine pesticides and hepatitis B markers | - N = 356 - Impact of organochlorine pesticides and hepatitis B on adverse events and whether benzonal therapy can counteract these effects - Conclusion – “Pathogenetic benzonal therapy with enterosorption in tuberculosis enhances therapeutical efficiency by 20-25% and reduces the incidence of side effects by 1.5-2 times.” | Other form of adverse event prevention |
| Onishchenko VV | Probl Tuberk 1990;(7):43-5 | Tisamide in the complex treatment of tuberculosis | - N = 145 - Impact of tisamide - Conclusion – “Tisamide, when prescribed to newly-discovered patients with destructive tuberculosis, accelerates recovery, by excluding the risk of a hepatotoxic action.” | Prevention of hepatotoxicity |
| Pravada NS | Antibiot Khimioter 2014;59(5-6):15-9 | Meglumine acridonacetate and complex therapy of patients with newly identified advanced pulmonary tuberculosis | - Sample size not provided - Impact of meglumine acridonacetate (cycloferon) - Conclusion – “The use of cycloferon normalized the number of gamma interferon receptors, increased the gamma interferon serum levels, reduced the incidence of the side effects (liver damage) due to the use of TB drugs.” | Prevention by immunomodulatory therapy |
| Savula MM | Probl Tuberk 1993;(5):18-20 | Chemotherapy of destructive pulmonary tuberculosis with antioxidants and antihypoxic agents | - N = 155 - Impact of antioxidants on TB treatment - Conclusion – “The intensive chemotherapy promoted a decrease in duration of bacterial discharge, destruction discontimation, cavernous healing, reduced incidence rates of side effects.” | Other form of adverse event prevention |
| Shakya R | Kathmandu Univ Med J (KUMJ) Jan-Mar 2005;3(1):45-9 | Management of antitubercular drugs-induced hepatotoxicity and therapy reintroduction strategy in a TB clinic of Nepal | - N = 4 - Use of a re-challenge protocol following recovery from drug-induced liver injury - Conclusion – “The recurrence of hepatotoxicity is rare if reintroduction in done in a well planned manner.” | Treatment of hepatoxicity |
| Shevyreva EV | Antibiot Khimioter 2012;57(7-8):31-7 | Remaxol hepatoprotective therapy of patients with tuberculosis and HIV infection in day unit of tuberculosis dispensary | - Sample size not provided - Impact of ramaxol on drug-induced liver injury - Conclusion – “The use of remaxol…significantly improved the biochemical indices and lowered the level of the cytolytic and cholestatic syndromes.” | Prevention of hepatotoxicity |
| Shmelev EI | Probl Tuberk 1996;(6):57-60 | Correction of bronchial obstructive syndrome and antituberculous drugs-induced eosinophilia in patients with pulmonary tuberculosis by using plasmapheresis | - N = 70 - Impact of plasmapheresis on patients with adverse events associated with eosinophilia - Conclusion – “Plasmapheresis as a means for correcting drug-induced eosinophilia and bronchial obstructive syndrome was found to be more effective than drug therapy…” | Treatment of hypersensitivity |
| Sukhanov DS | Antibiot Khimioter 2008;53(5-6):51-7 | Correction of adverse reactions in antimicrobial therapy of respiratory tract tuberculosis | - Sample size not provided - Impact of ramaxonol - Conclusion – “The positive influence of remaxol on the signs of the drug hepatotoxicity and adaptation reactions was shown…” | Treatment of hepatoxicity |
| Xu, W.-Z | Chinese Traditional and Herbal Drugs 2014;45(22):3308-3310 | Clinical effect and safety of Feilaoling preparation combined with conventional therapy in treatment of pulmonary tuberculosis | - N = 120 - Impact of a traditional Chinese prescription that can improve immune function - Conclusion – “Feilaoling preparation has a reliable effect on promotion lesions absorption and reducing the side effects of Western anti-tuberculosis, with certain clinical value.” | Other form of adverse event prevention |
| Yu D | Zhonghua Jie He He Hu Xi Za Zhi Oct 2001;24(10):608-10 | Clinical research of pasinizid on retreated sputum positive pulmonary tuberculosis in senilities | - N = 116 - Impact of pasinizid - Conclusion – “The effect of pasinizid on senile retreated sputum positive pulmonary tuberculosis was better than HP and SH regimen. Due to its lower rates of side effects, it is recommended for clinical usage.” | Alternative to isoniazid |
| Zhukova EM | Probl Tuberk 1995;(3):25-7 | Tactivin in combined treatment of patients with pulmonary tuberculosis associated with gastrointestinal diseases | - N = 101 - Impact of an immunomodulator, tactivin - Conclusion – “Healing of the destruction foci occurred in 87% versus 70% in controls, side effects of antibacterial drugs and exacerbations of gastrointestinal diseases arose 2-2.5 times less frequently.” | Prevention by immunomodulatory therapy |
| Not listed | Antibiot Khimioter 2013;58(3-4):33-6 | Reamberin in tuberculosis chemotherapy and its effect on liver function | - N = 257 - Impact of reamberin to prevent adverse events - Conclusion – “Adverse reactions (toxic, allergic) to the antituberculosis chemotherapeuties were 2.4 times less frequent in the patients additionally treated with reamberin.” | Other form of adverse event prevention |
