# Supplementary material for: A scoping review of interventions to prevent and treat adverse events during treatment of rifampin-susceptible tuberculosis
Source: PLoS One. 2025 Dec 26;20(12):e0339354. doi: 10.1371/journal.pone.0339354 (PMC12742745; doi:10.1371/journal.pone.0339354)
Supplement: S4 Table — (DOCX) [file pone.0339354.s004.docx]

S4 Table. Characteristics of the papers included in the scoping review (see the published paper for full citations

| First author (reference) | Title | Single site or multicenter study? | Country of enrollment | Study type | Study category, per the framework | Sample size per arm or cohort size | Brief summary of the study outcome |
| --- | --- | --- | --- | --- | --- | --- | --- |
| Abbaspour | Managing Hepatotoxicity Caused by Anti-tuberculosis Drugs: A Comparative Study of Approaches. | Single-site | Iran | Retrospective cohort | Treatment of hepatotoxicity | 77 | Similar outcomes with sequential and graded sequential re-challenge; 28 patients were not rechallenged with pyrazinamide |
| Adewole | Atorvastatin improve sputum conversion and chest x-ray severity score | Multicenter | Nigeria | RCT | Prevention of hepatoxicity | 75 | Increased muscle pain in atorvastatin group, increased neuropathy symptoms in controls; no difference in hepatotoxicity |
| Agal | Monitoring and management of antituberculosis drug induced hepatotoxicity | Single-site | India | Prospective cohort | Treatment of hepatotoxicity | 200 | Liver test monitoring (weekly x 1 month, then every 2 weeks for 2 months and then monthly) prevented icteric hepatitis |
| Akkahadsee | Systematic review and network meta-analysis of efficacy and safety of interventions for preventing anti-tuberculosis drug induced liver injury | Not applicable |  | Systematic review / meta-analysis | Prevention of hepatoxicity | 3423 | Turmeric and *Tinospora cordifolia* and N-acetyl cycteine significantly reduced hepatotoxicity; silymarin was not effective; polyherbal preparations resulted in lower lab values, but sample sizes were limited |
| Amagon | Methionine and vitamin B-complex ameliorate antitubercular drugs-induced toxicity in exposed patients | Multicenter | Nigeria | RCT | Prevention of hepatoxicity | 136 | Improvements in a laboratory and clinical outcomes: liver tests at 2 months (ALT, AST, total bilirubin), but not at 6 months; decreased adverse drug reactions (32% vs. 56%), including hepatotoxicity |
| Azuma | NAT2 genotype guided regimen reduces isoniazid-induced liver injury and early treatment failure in the 6-month four-drug standard treatment of tuberculosis: a randomized controlled trial for pharmacogenetics-based therapy. | Multicenter | Japan | RCT | Change in INH use/dose | 86 | Decrease in combined unfavorable outcomes at 2 months (17% vs. 48%), decreases in both components of the primary endpoint. Specifically, the decrease in INH-related hepatotoxicity was among slow-acetylators. Definition of liver injury was non-standard (ALT > twice normal) |
| Batbold | Double-blind, placebo-controlled, 1:1 randomized Phase III clinical trial of Immunoxel honey lozenges as an adjunct immunotherapy in 269 patients with pulmonary tuberculosis | Multicenter | Mongolia, Ukraine | RCT | Other form of prevention | 135 | Improvements in time to sputum culture clearance, body weight, duration of fever, liver and hematologic measures. No mention of clinical hepatotoxicity |
| Bermingham | Practical management of suspected hypersensitivity reactions to anti-tuberculosis drugs | Not applicable |  | Systematic review / meta-analysis | Protocol for re-challenge for hypersensitivity | 64 | Most (> 80%) of patients were able to continue rifampin |
| Borisova * | The use of fenazid in patients with pulmonary tuberculosis with poor isoniazid tolerance | Single-site | Russia | Prospective cohort | Other treatment of adverse event - other | 25 | No patients on fenazid developed hepatotoxicity, and all patients were able to complete therapy with neurological adverse events. |
| Bose | Intermittent versus daily therapy for treating tuberculosis in children | Not applicable |  | Systematic review / meta-analysis | Change in dosing frequency | 563 | No difference in adverse events; trend toward decreased treatment-limiting adverse events with intermittent therapy (0.4, 95% CI 0.06-2.6) |
| Bunkar | Add-on prednisolone in the management of cervical lymph node tuberculosis | Single-site | India | RCT | Corticosteroid or other immunomodulator | 60 | Improvements in symptom relief at 2 and 6 months, decreased complications (abscess, sinus new adenopathy). Increase in gastrointestinal events with prednisone. |
| Butov | Efficacy and safety of quercetin and polyvinylpyrrolidone in treatment of patients with newly diagnosed destructive pulmonary tuberculosis in comparison with standard antimycobacterial therapy | Single-site | Ukraine | Prospective cohort | Other form of prevention | 124 | Improvements in weight gain, inflammatory markers, and respiratory symptoms; decreased treatment-related adverse events (29% vs. 9%) |
| Canete | Tuberculous pleural effusion: experience with six months of treatment with isoniazid and rifampicin | Single-site | Spain | Prospective cohort | Change in PZA use | 130 | 15 did not complete treatment, of those who did all 115 had clinical resolution and did not have relapse. 5 had "symptomatic hepatitis" |
| Chaitra | Antituberculosis drug induced liver injury: clinical profile and outcome of various reintroduction regimens | Single-site | India | Prospective cohort | Treatment of hepatotoxicity | 50 | Better outcomes with sequential re-challenge |
| Chang | Standard anti-tuberculosis treatment and hepatotoxicity: do dosing schedules matter | Single-site | China | Retrospective cohort | Change in dosing frequency | 288 | No significant association between dosing frequency and hepatotoxicity, including in models that did and did not include alcohol use and Hepatitis B surface antigen positivity |
| Chang | Hepatotoxicity of pyrazinamide: cohort and case-control analyses | Single-site | China | Retrospective cohort | Change in PZA use | 3007 | Significant relationship between extended pyrazinamide use and occurrence of hepatotoxicity after 12 weeks of treatment (OR 2.8, 1.4-5.9) |
| Chen | Effectiveness of Prophylactic Use of Hepatoprotectants for Tuberculosis Drug-Induced Liver Injury: A Population-Based Cohort Analysis Involving 6,743 Chinese Patients | Single-site | China | Retrospective cohort | Prevention of hepatoxicity | 6,473 | No evidence of decreased hepatotoxicity events in persons on putative hepatoprotectants |
| Chen * | Clinical study of deoxyribonucleotidum for adjuvant treatment of pulmonary tuberculosis with hepatic lesion | Single-site | China | RCT | Treatment of hepatotoxicity | 40 | Patients were enrolled if ALT was > 40 during TB treatment. They were evidently continued on HRZ and half received the IV treatment for 4 weeks; not clear that there were any differences in clinical adverse events |
| Chien | Safety of rifabutin replacing rifampicin in the treatment of tuberculosis: a single-centre retrospective cohort study | Single-site | Taiwan | Retrospective cohort | Change in the rifamycin | 221 | 158 patients (72%) tolerated rifabutin; higher recurrence frequency among patients who had rifampin-related arthralgia, skin rash, or cholestasis, as well as females and those with chronic viral hepatitis |
| Chu | Role of bicyclol in preventing drug-induced liver injury in tuberculosis patients with liver disease | Multicenter | China | RCT | Prevention of hepatoxicity | 116 | Mild to severe liver injury in the test and control groups was respectively 8.6% {n = 10) and 18.4% {n = 21, P = 0.028). Fewer patients in the treatment arm interrupted TB treatment (1.7% vs. 7.9%). |
| Chuchottaworn | Adverse drug reactions and outcome of short course anti-tuberculosis drugs between single daily dose and split drug dose (BID) in pulmonary tuberculosis | Single-site | Thailand | RCT | Change in dosing frequency | 61 | Participants on twice-daily treatment regimens had a lower frequency of adverse drug reactions (52.5% vs. 62.3%) and somewhat higher frequency of cure (84.6% vs. 72.9%), but these differences did not reach statistical significance. |
| Critchley | Adjunctive steroid therapy for managing pulmonary tuberculosis | Not applicable |  | Systematic review / meta-analysis | Corticosteroid or other immunomodulator | 3816 | No differences seen in adverse events, though differences in reporting limited cross-study comparisons |
| Cross | Rosuvastatin adjunctive therapy for rifampicin-susceptible pulmonary tuberculosis: a phase 2b, randomised, open-label, multicentre trial | Multicenter | Philippines, Viet Nam, and Uganda | RCT | Prevention of hepatoxicity | 68 | No difference in adverse events, including hepatotoxicity |
| Dange | Efficacy of stimuliv, an indigenous compound formulation, against hepatotoxicity of antitubercular drugs--a double blind study | Single-site | India | RCT | Prevention of hepatoxicity | 62 | Decreased frequency of symptomatic and subclinical hepatitis (as determined by liver tests): 8% vs. 27%; all patients recovered and were able to resume TB treatment (HRZ) |
| Davies | Rifabutin for treating pulmonary tuberculosis | Not applicable |  | Systematic review / meta-analysis | Change in the rifamycin | 924 | No difference in adverse events, though a trend toward higher adverse events with higher doses of rifabutin |
| De Castro | Treatment With a Three-Drug Regimen for Pulmonary Tuberculosis Based on Rapid Molecular Detection of Isoniazid Resistance: A Noninferiority Randomized Trial (FAST-TB) | Multicenter | France | RCT | Change in ethambutol use | 102 | No difference in TB treatment outcomes or grade 3+ adverse events; ethambutol toxicity was suspected in 2 patients in the control arm and led to ethambutol discontinuation. |
| Deanasa | Efficacy and safety rifapentine-containing regimen for drug sensitive tuberculosis: Systematic review and meta-analysis | Not applicable |  | Systematic review / meta-analysis | Change in the rifamycin | 3655 | No difference in efficacy or adverse events between rifampin and rifapentine |
| Donovan | Adjunctive Dexamethasone for Tuberculous Meningitis in HIV-Positive Adults | Multicenter | Vietnam, Indonesia | RCT | Corticosteroid or other immunomodulator | 260 | Frequency of clinical and laboratory grade 3+ adverse events and the frequency of adverse events leading to discontinuation of TB or HIV treatments were similar between study arms, except that high ALT was more frequent in the dexamethasone arm (13.7% vs. 7.8%) |
| Du | Efficacy and safety of bicyclol for treating patients with antituberculosis drug-induced liver injury | Multicenter | China | RCT | Treatment of hepatotoxicity | 49 | ALT values normalized more rapidly in the low-dose and high-dose bicyclol arms than in the control arm; no difference in clinical adverse events; no mention of ability to re-introduce TB drugs |
| Dutt | Tuberculous pleural effusion: experience with six months of treatment with isoniazid and rifampicin | Single-site | United States | Prospective cohort | Change in PZA use | 198 | Major side effects in 3 patients (hepatitis in two, thrombocytopenia in one). 10 patients had minor side effects resulting in medication changes. |
| Emrani | Ginger for Prevention of Antituberculosis-induced Gastrointestinal Adverse Reactions Including Hepatotoxicity: A Randomized Pilot Clinical Trial | Single-site | Iran | RCT | Prevention of GI symptoms | 30 | Nausea was less common among participants on ginger (70% vs. 90%, p = 0.05). Trends toward less vomiting and hepatotoxicity in participants on ginger |
| Gallardo | Fixed-dose combinations of drugs versus single-drug formulations for treating pulmonary tuber | Not applicable |  | Systematic review / meta-analysis | Other change in the regimen | 5824 | No difference in serious adverse events (RR 1.45, 0.90 to 2.33), death (RR 0.96, 0.67 to 1.39), or treatment discontinuation due to adverse events (RR 0.96, 0.56 to 1.66). |
| Gao | Adjunctive therapy with interferon-gamma for the treatment of pulmonary tuberculosis: A systematic review | Not applicable |  | Systematic review / meta-analysis | Corticosteroid or other immunomodulator | 610 | No difference in adverse events (RR - 0.89 [0.59-1.35]) |
| García-Rodríguez | Long-term efficacy of 6-month therapy with isoniazid and rifampin compared with isoniazid, rifampin, and pyrazinamide treatment for pleural tuberculosis | Single-site | Spain | Retrospective cohort | Change in PZA use | 100 | All patients were cured and there were no relapses; fewer adverse events with 6HR vs. HRZ (15.3% vs. 33%); lower risk of hepatotoxicity (5% vs. 10.9%) |
| Gong | Comparative effectiveness of glycyrrhizic acid preparations aimed at preventing and treating anti-tuberculosis drug-induced liver injury: A network meta-analysis of 97 randomized controlled trials | Not applicable |  | Systematic review / meta-analysis | Prevention of hepatoxicity | 10923: 5762 (prevention), 5161 (treatment of DILI) | Prevention: four preparations were effective in preventing hepatotoxicity (RR 0.26-0.33) Treatment: Multiple different preparations were effective |
| Grace | Shortened treatment regimens versus the standard regimen for drug-sensitive pulmonary tuberculosis | Not applicable |  | Systematic review / meta-analysis | Other change in the regimen | 5825 | Very similar rates of adverse events with either moxifloxacin or gatifloxacin compared to control |
| Hagiwara | Safety of pyrazinamide-including regimen in late elderly patients with pulmonary tuberculosis: A prospective randomized open-label study | Single-site | Japan | RCT | Change in PZA use | 45 | Higher rate of discontinuation due to hepatotoxicity in the HRE group (15.6% vs. 9.1%), but this did not reach statistical significance; no difference in liver injury > 2.5 x ULN; higher death rate with pyrazinamide (10 vs. 3, p = 0.032) |
| Hakimizad | The Effect of acetyl-L-carnitine, Alpha-lipoic Acid, and Coenzyme Q10 Combination in Preventing Anti-tuberculosis Drug-induced Hepatotoxicity: A Randomized, Double-blind, Placebo-controlled Clinical Trial | Multicenter | Iran | RCT | Prevention of hepatoxicity | 44 | Lower incidence of hepatotoxicity in experimental arm (6.8% vs. 25.6%); most cases were mild |
| Hayford | The effects of anti-inflammatory agents as host-directed adjunct treatment of tuberculosis in humans: a systematic review and meta-analysis | Not applicable |  | Systematic review / meta-analysis | Other form of prevention | 2540 | Faster sputum smear conversion with vitamin D, but no difference in sputum culture conversion at 2 months; no differences in adverse events |
| Horita | Currently Used Low-Dose Pyrazinamide Does Not Increase Liver-Injury in the First Two Months of Tuberculosis Treatment | Multicenter | Japan | Retrospective cohort | Change in PZA use | 75 - HRE, 308 - HRZE | Increased risk of hepatotoxicity among patients treated with HRE, including in models using propensity matching for controls |
| Horne | Experience with rifabutin replacing rifampin in the treatment of tuberculosis | Single-site | United States | Retrospective cohort | Change in the rifamycin | 100 | 81% completed therapy with rifabutin (81% of those who had rifampin toxicity) |
| Hsieh | Efficacy of acupressure to prevent adverse reactions to anti-tuberculosis drugs: randomized controlled trials | Multicenter | Taiwan | RCT | Other form of prevention | 16 | Fewer adverse skin reactions in the group who received acuppressure using anatomic points thought to help adverse reactions vs. sham acuppressure |
| Huang | The effect of statins on the risk of anti-tuberculosis drug-induced liver injury among patients with active tuberculosis: A cohort study | Single-site | Taiwan | Retrospective cohort | Prevention of hepatoxicity | 1312 (50 TB patients received statins) | Hepatotoxicity less common among statin users (4% vs. 15.1%) as was severe DILI (2% vs 11%), including in multivariate models |
| Jo | Early discontinuation of ethambutol in pulmonary tuberculosis treatment based on results of the GenoType MTBDRPlus assay: A prospective, multicenter, noninferiority randomized trial in South Korea | Multicenter | Korea | RCT | Change in ethambutol use | 247 | No difference in treatment success (94% vs. 94%), no difference in adverse drug reactions (10% in each group), lower use of ophthalmologic exams in the genotypic resistance group |
| Johnson | Shortening treatment in adults with noncavitary tuberculosis and 2-month culture conversion | Multicenter | Uganda, Brazil, Phillipines | RCT | Other change in the regimen | 197 | No difference in adverse events |
| Kamal | Prospective, Open Labelled, Randomised, Parallel Group Study To Evaluate The Efficacy And Safety Of Metformin Add-On Therapy To Standard ATT In Newly Diagnosed Pulmonary Tuberculosis Patients | Single-site | India | RCT | Other form of prevention | 50 | No differences in clinical or laboratory adverse events |
| Kaneko * | Drug-induced hepatotoxicity caused by anti-tuberculosis drugs in tuberculosis patients complicated with chronic hepatitis | Single-site | Japan | Retrospective cohort | Change in PZA use | 107 | Patients treated with HRZ had a higher incidence of hepatotoxicity than those treated with HR (22% vs. 7%, p < 0.05). All 13 patients who developed hepatotoxicity on HRZ completed therapy, 9 stopped pyrazinamide |
| Katikova * | Use of the plant hepatoprotector Galstena tuberculostatics-induced hepatic lesions: experimental and clinical study | Single-site | Russia | Prospective cohort | Treatment of hepatotoxicity | 25 | Clinical (fatigue, abdominal pain, anorexia, weight loss) and laboratory parameters (ALT, bilirubin) improved |
| Kobashi * | Desensitization therapy for allergic reactions of antituberculous drugs--evaluation of desensitization therapy according to the guideline of the Japanese Society for Tuberculosis | Multicenter | Japan | Prospective cohort | Protocol for re-challenge for hypersensitivity | 19 | Successful in 16 patients (84%); the failures developed recurrent skin rash, but no severe adverse reactions |
| Kolomoiets * | The effect of the preparation Wobenzym on the antioxidant protection indices and on the functional-morphological properties of the erythrocytes in a toxic lesion of the liver | Single-site | Ukraine | RCT | Treatment of hepatotoxicity | 30 | More rapid normalization of liver enzymes; not clear that there was a difference in clinical adverse events |
| Kumar | Incidence and risk factors of antituberculosis drug-induced liver injury in India: a systematic review and meta-analysis | Not applicable |  | Systematic review / meta-analysis | Prevention of hepatoxicity | 12041 | Incidence of hepatotoxicity was 12.6 (9.9% using cut-off of > 5 x upper limit of normal); hepatotoxicity was higher with daily therapy (16.3%) than thrice-weekly therapy (3.5%) |
| Kura | Reintroducing antituberculosis therapy after Stevens-Johnson syndrome in human immunodeficiency virus-infected patients with tuberculosis: role of desensitization | Single-site | India | Prospective cohort | Protocol for re-challenge for hypersensitivity | 8 | 7 of 8 patients tolerated desensitization |
| Lee | Substitution of ethambutol with linezolid during the intensive phase of treatment of pulmonary tuberculosis: a prospective, multicentre, randomised, open-label, phase 2 trial | Multicenter | Korea | RCT | Change in ethambutol use | 143 | No differences in grade 3+ adverse events and no cases of optic neuritis; very few hematologic events and no trend toward adverse events among linezolid-treated patients |
| Lee | The Effectiveness and Safety of Fluoroquinolone-Containing Regimen as a First-Line Treatment for Drug-Sensitive Pulmonary Tuberculosis: A Systematic Review and Meta-Analysis | Not applicable |  | Systematic review / meta-analysis | Other change in the regimen | 6334 | Increase in total adverse events in patients treated with fluoroquinolones (OR 1.84, 1.46-2.31). Replacement of ethambutol was associated with increased adverse events; replacement of isoniazid was not. No significant difference in hepatotoxicity, drug rash, serious adverse events, or fever, Gastrointestinal adverse events, dizziness, and joint pains were more frequent with fluoroquinolones. |
| Lehloenya | Therapeutic trial of rifabutin after rifampicin-associated dress syndrome in tuberculosis-human immunodeficiency virus coinfected patients | Single-site | South Africa | Prospective cohort | Change in the rifamycin | 6 | All 6 tolerated rifabutin |
| Lehloenya | Diagnostic patch testing following tuberculosis-associated cutaneous adverse drug reactions induces systemic reactions in HIV-infected persons | Single-site | South Africa | Prospective cohort | Protocol for re-challenge for hypersensitivity | 11 | Patch testing results in systemic reactions in 10/11 patients (2 severe, nonlife-threatening) |
| Lehloenya | Outcomes of reintroducing anti-tuberculosis drugs following cutaneous adverse drug reactions | Single-site | South Africa | Retrospective cohort | Protocol for re-challenge for hypersensitivity | 46 | 23 (50%) had reactions to re-challenge; factors associated with unsuccessful re-challenge were initial treatment for TB and re-challenge with rifampin |
| Lian | Prophylactic antiviral treatment reduces the incidence of liver failure among patients coinfected with Mycobacterium tuberculosis and hepatitis B virus | Single-site | China | Retrospective cohort | Prevention of hepatoxicity | 90 | Treatment of Hepatitis B was associated with a lower risk of liver failure and death (OR 0.033 (0.007-0.154). Hepatitis B viral load was associated with an increased risk of liver failure. |
| Lin | Efficacy of proprietary Lactobacillus casei for anti-tuberculosis associated gastrointestinal adverse reactions in adult patients: a randomized, open-label, dose-response trial | Single-site | China | RCT | Prevention of GI symptoms | 131 | Decrease in total gastrointestinal side effects (29.4%, 37.6%, 50% [control]); specific symptoms decreased - vomiting, anorexia); there was also a decrease in the GI adverse event burden over time (fewer days of symptoms) |
| Lin | Efficacy and safety of short-term chemotherapy for patients with spinal tuberculosis undergoing surgery in Chinese population: a meta-analysis | Not applicable | China | Systematic review / meta-analysis | Change in PZA use | 842 | 6-month drug treatment regimens had higher clinical cure (not statistically significant) and lower side effects (OR 0.37, 0.24-0.48). Longer regimens included PZA for > 2 months |
| Lui | Antiviral Therapy for Hepatitis B Prevents Liver Injury in Patients with Tuberculosis and Hepatitis B Coinfection | Single-site | China | Retrospective cohort | Prevention of hepatoxicity | 3698 with TB-HepB; 488 on antiviral therapy | In an analysis adjusted for propensity score, patients on antiviral therapy had a lower risk of hospitalization for hepatotoxicity (aHR 0.44, 0.26-0.72) and lower risk of the composite of hospitalization or ALT > 3 x upper limit of normal; no difference in liver-related death |
| Lysov * | Adverse neurotoxic reactions of chemotherapy for tuberculosis and their treatment | Single-site | Russia | Prospective cohort | Other treatment of adverse event - other | 21 | More rapid recovery from neurological symptoms, more patients were able to restart isoniazid (91% vs. 42%) |
| Maddahi | The efficacy of Jujube syrup on the prevention of drug-induced hepatotoxicity in pulmonary tuberculosis patients: A pilot randomized double-blind placebo-controlled clinical trial | Multicenter | Iran | RCT | Prevention of hepatoxicity | 17 | Fewer patient on Jujube syrup had hepatotoxicity (3, 0) - not statistically significant |
| Magula * | Effect of silymarine and Fumaria alkaloids in the prophylaxis of drug-induced liver injury during antituberculotic treatment | Single-site | Slovakia | Prospective cohort | Prevention of hepatoxicity | 29 | Reductions of hepatotoxicity, but not statistically significant |
| Mahani | Antiemetic activities of indonesian stingless bee propolis on emetic induced by anti-tuberculosis drugs | Single-site | Indonesia | RCT | Prevention of GI symptoms | 17 | Greater decrease in nausea at week 24 in the two intervention arms (no statistical test provided) |
| Mai | A randomised double blind placebo controlled phase 2 trial of adjunctive aspirin for tuberculous meningitis in HIV-uninfected adults | Single-site | Vietnam | RCT | Corticosteroid or other immunomodulator | 40 | No statistically significant difference in grade 3+ events, though a trend toward increased adverse events in the low-dose aspirin arm |
| Makharia | Intermittent Directly Observed Therapy for Abdominal Tuberculosis: A Multicenter Randomized Controlled Trial Comparing 6 Months Versus 9 Months of Therapy | Multicenter | India | RCT | Other change in the regimen | 99 | No difference is side effects (21.3% with 6 months, 18.2% with 9 months) |
| Marjani | Evaluation of Silymarin for management of anti-tuberculosis drug induced liver injury: A randomized clinical trial | Single-site | Iran | RCT | Treatment of hepatotoxicity | 28 | No differences in any of the endpoints |
| Meintjes | Prednisone for the prevention of paradoxical tuberculosis-associated IRIS | Single-site | South Africa | RCT | Corticosteroid or other immunomodulator | 120 | Decrease in IRIS events (32.5% vs. 46.7%), grade 3 clinical events; trend toward decreases in hospitalization, composite including hepatotoxicity, and discontinuation of TB treatment or antiretroviral treatment; no difference in death |
| Meintjes | Randomized placebo-controlled trial of prednisone for paradoxical tuberculosis-associated immune reconstitution inflammatory syndrome | Single-site | South Africa | RCT | Corticosteroid or other immunomodulator | 55 | Decrease in the primary endpoint; improvement in IRIS symptoms and quality of life, greater improvement in chest radiograph |
| Meng | The role of vitamin D in the prevention and treatment of tuberculosis: a meta-analysis of randomized controlled trials | Not applicable |  | Systematic review / meta-analysis | Other form of prevention | 15,586 | No impact on sputum culture conversion, adverse events (RR 0.92, 0.71-1.14), or death; improvement in symptom score at 8 weeks |
| Millard | A study of the optimisation of tuberculosis therapy. Chapter 2: Systematic review and meta-analysis of the impact of inclusion, dose and duration of pyrazinamide in eficacy and safety outcomes in tuberculosis | Not applicable |  | Systematic review / meta-analysis | Change in PZA use | 6,414 | Impacts of pyrazinamide: increased total adverse events (RR 1.7, 1.33-2.17), regimen changes 1.89, 1.02-3.49); no increase in hepatotoxicity (0.81, 0.43-1.52), all-cause mortality, or TB mortality. Longer duration of pyrazinamide: increase hepatotoxicity (2.24, 0.32-15.81) |
| Misra | Role of aspirin in tuberculous meningitis: a randomized open label placebo controlled trial | Single-site | India | RCT | Corticosteroid or other immunomodulator | 59 | Decreased stroke by MRI (43.3%) in the placebo and 24.2%) in the aspirin group; decreased mortality, improved functional outcome, no difference in side effects |
| Misra | Standard versus sequential anti-tubercular treatment in patients with tuberculous meningitis: a randomized controlled trial | Single-site | India | RCT | Prevention of hepatoxicity | 40 | Decreased hepatotoxicity with graded, sequential initiation (20 vs. 42.5%), lower inpatient mortality and days hospitalized, decreased paradoxical worsening, no difference in other adverse events |
| Miwa | Drug lymphocyte stimulation test is not useful for side effects of anti-tuberculosis drugs despite its timing | Single-site | Japan | RCT | Protocol for re-challenge for hypersensitivity | 16 | Poor sensitivity of drug lymphocyte stimulation tests to predict results of re-challenge |
| Modi | Spectrum of anti tubercular therapy induced cutaneous adverse drug reactions and its management through rechallenge: A prospective study at a Tertiary Care Centre | Single-site | India | Prospective cohort | Protocol for re-challenge for hypersensitivity | 56 | Ethambutol was the most common implicated drug > pyrazinamide > isoniazid > rifampin, levofloxacin |
| Moosa | Rechallenge after anti-tuberculosis drug-induced liver injury in a high HIV prevalence cohort | Multicenter | South Africa | Prospective cohort | Treatment of hepatotoxicity | 79 | Positive re-challenge in 14 (18%) - associated with female sex, first episode of TB, and pyrazinamide (one patient died of rash, jaundice, and acute kidney injury after re-challenge with pyrazinamide) |
| Morán-Mariños | DRESS syndrome and tuberculosis: Implementation of a desensitization and re-desensitization protocol to recover antituberculosis drugs in a case series at a specialized TB Unit in Lima, Peru | Single-site | Peru | Retrospective cohort | Protocol for re-challenge for hypersensitivity | 18 | 7 received rapid desensitization, achieving a success rate of 63.6%, while only 2 of the patients (28.5%) successfully underwent slow desensitization. |
| Oh | Reintroduction of Antituberculous Drugs in Patients with Antituberculous Drug-Related Drug Reaction with Eosinophilia and Systemic Symptoms | Multicenter | Korea | Retrospective cohort | Protocol for re-challenge for hypersensitivity | 29 | Desensitization (starting form very small doses seemed to be more successful than graded challenge (starting with a dose near the treatment dose) |
| Padmapriydarsini | Randomized Trial of Metformin With Anti-Tuberculosis Drugs for Early Sputum Conversion in Adults With Pulmonary Tuberculosis | Multicenter | India | RCT | Other form of prevention | 161 | Increased nausea/vomiting with metformin (30 vs 4, P < .001) |
| Pasipanodya | Clinical and Toxicodynamic Evidence that High-Dose Pyrazinamide Is Not More Hepatotoxic than the Low Doses Currently Used | Not applicable |  | Systematic review / meta-analysis | Change in PZA use | 13,000 | "…the frequency of hepatotoxicity and jaundice was less than 6% across all regimens tested and did not differ whether or not pyrazinamide was included in a regimen." "...hepatotoxicity occurred with more frequent dosing despite a slightly lower cumulative dose…" |
| Prasad | Corticosteroids for managing tuberculous meningitis | Not applicable |  | Systematic review / meta-analysis | Corticosteroid or other immunomodulator | 1337 | There was no difference between the groups in the incidence of adverse events, which included gastrointestinal bleeding, invasive bacterial infections, hyperglycemia, and liver dysfunction (RR 0.88, 0.76-1.17) |
| Reuter | Implementing a Substance-Use Screening and Intervention Program for People Living with Rifampicin-Resistant Tuberculosis: Pragmatic Experience from Khayelitsha, South Africa | Single-site | South Africa | Prospective cohort | Other form of prevention (details in the Comment variable | 103 | Increased loss to follow-up in persons with alcohol use disorder; no association between naltrexone use and treatment outcomes |
| Ryan | Corticosteroids for tuberculous pleurisy | Not applicable |  | Systematic review / meta-analysis | Corticosteroid or other immunomodulator | 590 | Adverse events leading to regimen discontinuation more common with corticosteroids (RR 2.78, 95% CI 1.11 to 6.94). Among HIV-positive patients, more cases of Kaposi's sarcoma with corticosteroids (6/99) than control (0/98) |
| Safe | Safety and efficacy of N-acetylcysteine in hospitalized patients with HIV-associated tuberculosis: An open-label, randomized, phase II trial (RIPENACTB Study) | Single-site | Brazil | RCT | Prevention of hepatoxicity | 25 | Increased nausea and hepatotoxicity in N-acetyl cysteine patients (p > 0.05) |
| Saigal | Safety of an ofloxacin-based antitubercular regimen for the treatment of tuberculosis in patients with underlying chronic liver disease: a preliminary report | Single-site | India | RCT | Change in PZA use | 16 | Hepatotoxicity more common with IRE than IZEOflox (26.6% vs. 0, p -0.043) |
| Saito | Effectiveness of hepatoprotective drugs for anti-tuberculosis drug-induced hepatotoxicity: a retrospective analysis | Single-site | Japan | Retrospective cohort | Treatment of hepatotoxicity | 66 | No difference in time to normalization of ALT or in peak ALT/AST |
| Sanabria-Cabrera | N-Acetylcysteine for the Management of Non-Acetaminophen Drug-Induced Liver Injury in Adults: A Systematic Review | Not applicable |  | Systematic review / meta-analysis | Prevention of hepatoxicity | 686 | Treatment: N-acetyl cysteine treatment shows an inconclusive effect in terms of overall survival, but did improve transplant-free survival. Prevention: N-acetyl cysteine reduced hepatotoxicity but had no effect on severity in patients exposed to TB drugs. |
| Santha | Split-drug regimens for the treatment of patients with sputum smear-positive pulmonary tuberculosis -a unique approach | Multicenter | India | RCT | Change in dosing frequency | 413 | Freedom from adverse events was improved with two split-drug regimens (Split 1 HR 0.81, p = 0.06; Split 2 HR 0.58, P=0.002)). Adverse events attributable to TB drugs: Split 1: 10%, Split - 2: 15%, daily: 14%; gastrointestinal adverse events 7% vs. 3% [both split arms], p = 0.01) |
| Shamaei | Recurrent Drug-Induced Hepatitis in Tuberculosis-Comparison of Two Drug Regimens | Single-site | India | Retrospective cohort | Change in PZA use | 135 | 21% on HREOflox had recurrent hepatotoxicity vs. 15% of HREZ |
| Shangguan } | Randomized control study of the use of faropenem for treating patients with pulmonary tuberculosis | Multicenter | China | RCT | Change in ethambutol use | 114 | Fewer side effects in the faropenem group (32% vs. 50%, p < 0.01); no difference in grade 3+ adverse events. Decrease in visual impairment in faropenem group (0 vs. 4.7%, p = 0.02). |
| Sharma | Six months versus nine months anti-tuberculous therapy for female genital tuberculosis: a randomized controlled trial | Single-site | India | RCT | Other change in the regimen | 88 | No trend regarding adverse events: 6 months - 33%; 9 months - 31% |
| Shin | Effectiveness of alcohol treatment interventions integrated into routine tuberculosis care in Tomsk, Russia | Single-site | Russia | RCT | Other form of prevention | 100 | Neither naltrexone no behavioral counseling decreased adverse events |
| Singanayagam | A comparison between two strategies for monitoring hepatic function during antituberculous therapy | Single-site | United Kingdom | Prospective cohort | Prevention of hepatoxicity | 288 (21 developed DILI) | “…American Thoracic Society algorithm had a sensitivity and specificity of 66.7 and 65.6%, respectively, for prediction of early and 22.2% and 63.7% for late drug-induced liver injury. The uniform monitoring policy had poor sensitivity but better specificity (22.2 and 82.1%) for prediction of late drug-induced liver injury.” |
| Siripassorn | Successful drug desensitization in patients with delayed-type allergic reactions to anti-tuberculosis drugs | Multicenter | Thailand | Retrospective cohort | Protocol for re-challenge for hypersensitivity | 13 | 79% had successful re-challenges, 63% among those with prior severe hypersensitivity (e.g. Stevens Johnson Syndrome, Drug-Related Drug Reaction with Eosinophilia and Systemic Symptoms [DRESS]) |
| Smadhi * | Allergy to anti-tuberculosis treatment: Place of reintroduction drug test | Single-site | Tunesia | Retrospective cohort | Protocol for re-challenge for hypersensitivity | 27 | 27 patients underwent re-challenge; 6 patients underwent rapid desensitization, 5 successfully |
| Soni | Risk of hepatitis with various reintroduction regimens of anti-tubercular therapy: a systematic review and network meta-analysis | Not applicable |  | Systematic review / meta-analysis | Treatment of hepatotoxicity | 577 | Incremental (graded dose escalation) better than sequential (OR 0.33, 0.033 1.7) and concomitant (OR 0.24, 0,017-1.2); no difference by rifampin or isoniazid first |
| Srinivasan | Salubrious effect of vitamin E supplementation on renal stone forming risk factors in urogenital tuberculosis patients | Single-site | India | Prospective cohort | Other form of prevention | 36 | Normal renal cell morphology among patients on vitamin E, decreased excretion of oxalate |
| Sukumaran | A study to evaluate the hepatoprotective effect of N- acetylcysteine on anti tuberculosis drug induced hepatotoxicity and quality of life | Single-site | India | RCT | Prevention of hepatoxicity | 19 | Between group analysis showed, significant reduction in ALT (p < 0.05) and AST (p < 0.05) in N-acetyl cysteine group at 4 weeks; no difference in clinical adverse events |
| Suzuki | Drug lymphocyte stimulation test in the diagnosis of adverse reactions to antituberculosis drugs | Single-site | Japan | Prospective cohort | Protocol for re-challenge for hypersensitivity | 261 | Drug lymphocyte stimulation test had poor sensitivity and marginal specificity in predicting response to re-challenge |
| Talebi | The Effectiveness of Silymarin in the Prevention of Anti-tuberculosis Drug-induced Hepatotoxicity: A Randomized Controlled Clinical Trial | Single-site | Iran | RCT | Prevention of hepatoxicity | 18 | ALT in the experimental group declined, while it increased in the control group; hepatotoxicity occurred in 0 and 2 patients, respectively |
| Taniguchi | Safety of pyrazinamide in elderly patients with tuberculosis in Japan: A nationwide cohort study | Multicenter | Japan | Retrospective cohort | Change in PZA use | 7,156 | In the propensity score matched analysis, no difference in in-hospital mortality (12.99% in HRE, 14% in HREZ); lower risk of overall adverse effects in HRE group (3.1% vs. 4.7%); no difference in hepatotoxicity (1.5% vs. 1.9%); results in the subgroup > 75 were similar |
| Tao | Prophylactic Therapy of Silymarin (Milk Thistle) on Antituberculosis Drug-Induced Liver Injury: A Meta-Analysis of Randomized Controlled Trials | Not applicable |  | Systematic review / meta-analysis | Prevention of hepatoxicity | 1198 | Silymarin reduced hepatotoxicity at week 4 [RR: 0.33, P = 0.008 (but not at weeks 2 and 8; no significant difference in adverse events [RR: 1.09, 95% CI (0.86, 1.39), P = 0.47] |
| Turkova | Shorter Treatment for Nonsevere Tuberculosis in African and Indian Children | Multicenter | Uganda, Zambia, South Africa, India | RCT | Other change in the regimen | 602 | No difference in TB outcomes; grade 3+ adverse events were similar; all adverse reactions except 3 occurred in the first 8 weeks of therapy |
| Ungo | Antituberculosis drug-induced hepatotoxicity. The role of hepatitis C virus and the human immunodeficiency virus | Single-site | United States | Retrospective cohort | Treatment of hepatotoxicity | 4 | All four patients treated for Hepatitis C were then able to tolerate re-introduction of INH and a rifamycin |
| Villamor | A trial of the effect of micronutrient supplementation on treatment outcome, T cell counts, morbidity, and mortality in adults with pulmonary tuberculosis | Multicenter | Tanzania | RCT | Other form of prevention | 444 | Micronutrients decreased the risk of neuropathy and genital ulcers |
| Wang | Hepatoprotective drugs for prevention of liver injury resulting from anti-tuberculosis treatment: A meta-analysis of cohort studies | Not applicable |  | Systematic review / meta-analysis | Prevention of hepatoxicity | 3589 | Use of hepatoprotective drugs associated with lower risk of hepatotoxicity (relative risk = 0.39, 95% confidence interval [CI]: 0.28–0.53, p < 0.001). In subgroup analyses, protection was noted for mild and moderate hepatotoxicity and liver injury within 2–4 weeks (RR = 0.37, 95% CI 0.19–0.71). Hepatoprotective drugs also associated with decreased TB treatment regimen changes (RR = 0.58, 95% CI 0.34–0.97, p = 0.040) |
| Wardhan | A study of the oxidative stress and the role of antioxidants in ATT induced hepatotoxicity in tuberculosis patients | Single-site | India | RCT | Treatment of hepatotoxicity | 5 | No differences in clinical outcomes |
| Wu | Preventive use of hepatoprotectors yields limited efficacy on the liver toxicity of anti-tuberculosis agents in a large cohort of Chinese patients | Multicenter | China | Prospective cohort | Prevention of hepatoxicity | 4488 | Similar risk of hepatotoxicity with hepatoprotectants (adjusted HR 1.05 (95% CI, 0.71 to 1.57, P = 0.808); no difference by type of hepatoprotectant drug |
| Wu | Effect of scheduled monitoring of liver function during anti-Tuberculosis treatment in a retrospective cohort in China | Multicenter | China | Prospective cohort | Prevention of hepatoxicity | 4488 | Of 20 cases of hepatotoxicity in the scheduled monitoring group, none became icteric, 12% developed severe hepatotoxicity, 2% hospitalized, 20% had prolonged TB treatment, 0 died. Of 162 "passive detection cases", 5 became icteric, 17% developed severe hepatotoxicity, 11% hospitalized, 27% had prolonged TB treatment, 2 patients died of hepatotoxicity |
| Xiong | Lactobacillus casei Alleviated the Abnormal Increase of Cholestasis-Related Liver Indices During Tuberculosis Treatment: A Post Hoc Analysis of Randomized Controlled Trial | Single-site | China | RCT | Prevention of hepatoxicity | 108 | Incidence of liver injury during the intensive phase of tuberculosis treatment was 5.7%, 4.7%, and 3.5% for the control group, the low-dose group and the high-dose group (p = 0.738). |
| Xu | Pyrazinamide safety, efficacy, and dosing for treating drug-susceptible pulmonary tuberculosis: a phase 3, randomized, controlled clinical trial | Multicenter |  | RCT | Change in PZA use | 781 | Efficacy and safety outcome were associated with pyrazinamide exposure, suggesting that an unadjusted dose of 1000 mg per day might decrease adverse events |
| Xu | Is the Prophylactic Use of Hepatoprotectants Necessary in Anti-Tuberculosis Treatment? | Not applicable |  | Systematic review / meta-analysis | Prevention of hepatoxicity | 1227 | Treatment with hepatoprotective agents decreased the number of patients with hepatotoxicity compared to controls (RR 0.50, 95% CI [0.34, 0.73], p = 0.0004) |
| Yanqiu | Impact of hyperglycemia on tuberculosis treatment outcomes: a cohort study | Single-site | China | Prospective cohort | Other form of prevention | 791 | Patients with hyperglycemia had higher incidence of adverse events (vomiting, diarrhea, and constipation; no association between diabetes treatment and adverse events |
| Yazdani * | The effects of livercare tablet [Combination of milk thistle, dandelion, barberry, tumeric (Curcumin), and artichoke] in prevention of anti-tuberculosis drugs-induced hepatotoxicity: A randomized controlled clinical trial | Single-site | Iran | RCT | Prevention of hepatoxicity | 22 | 6/22 patients (27%) in the control group developed hepatotoxicity compared with 1/22 (5%) in the multiherbal group (p = 0.039) |
| Yeswanth | A prospective single-blinded study on the safety and efficacy of zinc supplementation in pulmonary tuberculosis | Single-site | India | RCT | Other form of prevention (details in the Comment variable | 40 | Trend toward decreased gastrointestinal symptoms among participants on zinc (p = 0.06, but a trend in the opposite direction for constipation |
| Yoon | Impact of pyrazinamide usage on serious adverse events in elderly tuberculosis patients: A multicenter cohort study | Multicenter | Korea | Retrospective cohort | Change in PZA use | 1204 | Primary outcomes (Serious Adverse Event, medication interruption, or lost to follow-up) occurred significantly less frequently in with pyrazinamide; similar results for those 75 and older |
| Zaitzeva | Treatment of cavitary and infiltrating pulmonary tuberculosis with and without the immunomodulator Dzherelo | Single-site | Ukraine | Prospective cohort | Corticosteroid or other immunomodulator | 38 | No differences in clinical outcomes |
| Zhu | Baseline HBV load increases the risk of anti-tuberculous drug-induced hepatitis flares in patients with tuberculosis | Single-site | China | Prospective cohort | Prevention of hepatoxicity | 87 | Antiviral treatment for Hepatitis B was associated with a lower risk of liver failure (OR 0.027, 0.076 to 0.96); high Hepatitis B viral loads were associated with liver dysfunction episodes. |

* Paper translated using Chat GPT
