# Supplementary material for: A scoping review of interventions to prevent and treat adverse events during treatment of rifampin-susceptible tuberculosis
Source: PLoS One. 2025 Dec 26;20(12):e0339354. doi: 10.1371/journal.pone.0339354 (PMC12742745; doi:10.1371/journal.pone.0339354)
Supplement: S5 Table — (DOCX) [file pone.0339354.s005.docx]

S5 Table. Randomized trials having sample sizes < 50 patients per arm

| First author | Title | Sample size per arm |
| --- | --- | --- |
| Wardhan | A study of the oxidative stress and the role of antioxidants in ATT induced hepatotoxicity in tuberculosis patients | 5 |
| Hsieh | Efficacy of acupressure to prevent adverse reactions to anti-tuberculosis drugs: randomized controlled trials | 16 |
| Miwa | Drug lymphocyte stimulation test is not useful for side effects of anti-tuberculosis drugs despite its timing | 16 |
| Saigal | Safety of an ofloxacin-based antitubercular regimen for the treatment of tuberculosis in patients with underlying chronic liver disease: a preliminary report | 16 |
| Mahani | Antiemetic activities of indonesian stingless bee propolis on emetic induced by anti-tuberculosis drugs | 17 |
| Maddahi | The efficacy of Jujube syrup on the prevention of drug-induced hepatotoxicity in pulmonary tuberculosis patients: A pilot randomized double-blind placebo-controlled clinical trial | 17 |
| Talebi | The Effectiveness of Silymarin in the Prevention of Anti-tuberculosis Drug-induced Hepatotoxicity: A Randomized Controlled Clinical Trial | 18 |
| Sukumaran | A study to evaluate the hepatoprotective effect of N- acetylcysteine on anti tuberculosis drug induced hepatotoxicity and quality of life | 19 |
| Yazdani | The effects of livercare tablet [Combination of milk thistle, dandelion, barberry, tumeric (Curcumin), and artichoke] in prevention of anti-tuberculosis drugs-induced hepatotoxicity: A randomized controlled clinical trial | 22 |
| Safe | Safety and efficacy of N-acetylcysteine in hospitalized patients with HIV-associated tuberculosis: An open-label, randomized, phase II trial (RIPENACTB Study) | 25 |
| Marjani | Evaluation of Silymarin for management of anti-tuberculosis drug induced liver injury: A randomized clinical trial | 28 |
| Emrani | Ginger for Prevention of Antituberculosis-induced Gastrointestinal Adverse Reactions Including Hepatotoxicity: A Randomized Pilot Clinical Trial | 30 |
| Kolomoiets | The effect of the preparation Wobenzym on the antioxidant protection indices and on the functional-morphological properties of the erythrocytes in a toxic lesion of the liver | 30 |
| Chen | Clinical study of deoxyribonucleotidum for adjuvant treatment of pulmonary tuberculosis with hepatic lesion | 40 |
| Mai | A randomised double blind placebo controlled phase 2 trial of adjunctive aspirin for tuberculous meningitis in HIV-uninfected adults | 40 |
| Misra | Standard versus sequential anti-tubercular treatment in patients with tuberculous meningitis: a randomized controlled trial | 40 |
| Yeswanth | A prospective single-blinded study on the safety and efficacy of zinc supplementation in pulmonary tuberculosis | 40 |
| Hakimizad } | The Effect of acetyl-L-carnitine, Alpha-lipoic Acid, and Coenzyme Q10 Combination in Preventing Anti-tuberculosis Drug-induced Hepatotoxicity: A Randomized, Double-blind, Placebo-controlled Clinical Trial | 44 |
| Hagiwara | Safety of pyrazinamide-including regimen in late elderly patients with pulmonary tuberculosis: A prospective randomized open-label study | 45 |
| Du | Efficacy and safety of bicyclol for treating patients with antituberculosis drug-induced liver injury | 49 |
