# Supplementary material for: A scoping review of interventions to prevent and treat adverse events during treatment of rifampin-susceptible tuberculosis
Source: PLoS One. 2025 Dec 26;20(12):e0339354. doi: 10.1371/journal.pone.0339354 (PMC12742745; doi:10.1371/journal.pone.0339354)
Supplement: S6 Table — (DOCX) [file pone.0339354.s006.docx]

S6 Table. Inclusion of persons < 18 years of age

| First author | Title | Involvement of children | Enrollment < 12 years | Enrollment 12-17 |
| --- | --- | --- | --- | --- |
| Agal | Monitoring and management of antituberculosis drug induced hepatotoxicity | Children as young as 5 years were included, but not quantitated | Y | Y |
| Bunkar | Add-on prednisolone in the management of cervical lymph node tuberculosis | Children were involved (mean age 27.5, std dev 13), but not quantified) | Y | Y |
| Canete | Tuberculous pleural effusion: experience with six months of treatment with isoniazid and rifampicin | Age range was 11-53, number of children/youth not quantified | Y | Y |
| García-Rodríguez | Long-term efficacy of 6-month therapy with isoniazid and rifampin compared with isoniazid, rifampin, and pyrazinamide treatment for pleural tuberculosis | Clearly included (mean age 33 + 18 years), though not quantified | Y | Y |
| Misra | Role of aspirin in tuberculous meningitis: a randomized open label placebo controlled trial | 6 participants were < 12 years old | Y | Y |
| Saigal | Safety of an ofloxacin-based antitubercular regimen for the treatment of tuberculosis in patients with underlying chronic liver disease: a preliminary report | Enrolled patients from 8-63 | Y | Y |
| Turkova | Shorter Treatment for Nonsevere Tuberculosis in African and Indian Children | Children < 16 were enrolled, median age 3.5 years | Y | Y |
| Misra | Standard versus sequential anti-tubercular treatment in patients with tuberculous meningitis: a randomized controlled trial | Patients enrolled were 15-75 | N | Y |
| Morán-Mariños | DRESS syndrome and tuberculosis: Implementation of a desensitization and re-desensitization protocol to recover antituberculosis drugs in a case series at a specialized TB Unit in Lima, Peru | Two adolescents enrolled (15, 17 years) | N | Y |
| Santha | Split-drug regimens for the treatment of patients with sputum smear-positive pulmonary tuberculosis -a unique approach | Children > 12 years old enrolled, but not quantitated | N | Y |
| Shamaei | Recurrent Drug-Induced Hepatitis in Tuberculosis-Comparison of Two Drug Regimens | 16 to 86 years old | N | Y |
| Smadhi | Allergy to anti-tuberculosis treatment: Place of reintroduction drug test | Average age was 37 years (17 to 82 years) | N | Y |
| Xu | Pyrazinamide safety, efficacy, and dosing for treating drug-susceptible pulmopnary tuberculosis: a phase 3, randomized, controlled clinical trial | Children older than 12 were included | N | Y |
| Zhu | Baseline HBV load increases the risk of anti-tuberculous drug-induced hepatitis flares in patients with tuberculosis | Mean age was 44.38 years (range, 17–80 years) | N | Y |
| Lehloenya | Diagnostic patch testing following tuberculosis-associated cutaneous adverse drug reactions induces systemic reactions in HIV-infected persons | 13- and 16-year-old were included | N | Y |
| Lian | Prophylactic antiviral treatment reduces the incidence of liver failure among patients coinfected with Mycobacterium tuberculosis and hepatitis B virus | Down to age 16 enrolled | N | Y |
| Makharia | Intermittent Directly Observed Therapy for Abdominal Tuberculosis: A Multicenter Randomized Controlled Trial Comparing 6 Months Versus 9 Months of Therapy | Enrolled patients 15-65 years | N | Y |
| Mahani | Antiemetic activities of indonesian stingless bee propolis on emetic induced by anti-tuberculosis drugs | Enrolled patients 12-45 years | N | Y |
| Taniguchi | Safety of pyrazinamide in elderly patients with tuberculosis in Japan: A nationwide cohort study | Persons < 15 eliminated from the cohort analysis | N | Y |
| Abbaspour | Managing Hepatotoxicity Caused by Anti-tuberculosis Drugs: A Comparative Study of Approaches. | 1 person between 0 and 20 years was included | NR | NR |
| Dutt | Tuberculous pleural effusion: experience with six months of treatment with isoniazid and rifampicin | 2 persons 0-19 years | NR | NR |
| Chang | Standard anti-tuberculosis treatment and hepatotoxicity: do dosing schedules matter | Not clear if children were involved | NR | NR |
| Chang | Hepatotoxicity of pyrazinamide: cohort and case-control analyses | Not clear if children were involved | NR | NR |
| Horne | Experience with rifabutin replacing rifampin in the treatment of tuberculosis | Not clear whether children were included; doubtful given mean age of 48 + 18) | NR | NR |
| Katikova | Use of the plant hepatoprotector Galstena tuberculostatics-induced hepatic lesions: experimental and clinical study | Involvement of children not reported | NR | NR |
| Lehloenya | Outcomes of reintroducing anti-tuberculosis drugs following cutaneous adverse drug reactions | Not clear whether children were included | NR | NR |
| Lui | Antiviral Therapy for Hepatitis B Prevents Liver Injury in Patients with Tuberculosis and Hepatitis B Coinfection | Not clear whether children were involved. | NR | NR |
| Saito | Effectiveness of hepatoprotective drugs for anti-tuberculosis drug-induced hepatotoxicity: a retrospective analysis | Not clear if children were involved | NR | NR |
| Srinivasan | Salubrious effect of vitamin E supplementation on renal stone forming risk factors in urogenital tuberculosis patients | Not clear if children were involved | NR | NR |
| Yazdani | The effects of livercare tablet [Combination of milk thistle, dandelion, barberry, tumeric (Curcumin), and artichoke] in prevention of anti-tuberculosis drugs-induced hepatotoxicity: A randomized controlled clinical trial | Persons 15-65 eligible, not clear whether any persons < 30 enrolled | NR | NR |

NR – not reported
