# Supplementary figures and images for: A scoping review of interventions to prevent and treat adverse events during treatment of rifampin-susceptible tuberculosis
Source: PLoS One. 2025 Dec 26;20(12):e0339354. doi: 10.1371/journal.pone.0339354 (PMC12742745; doi:10.1371/journal.pone.0339354)

S7 Fig. PRISMA flow chart for the search of clinical trials registries


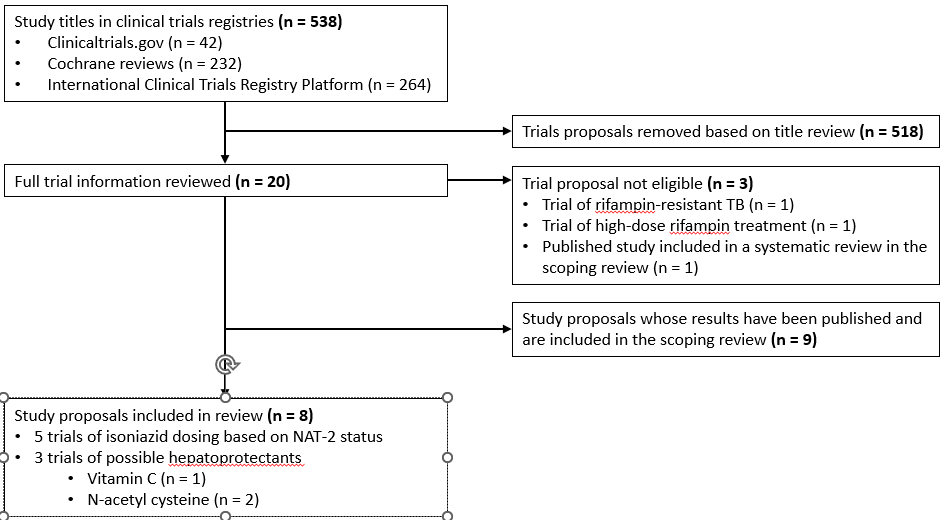

Supplement: S7 Fig — (DOCX) [file pone.0339354.s007.docx]
