# Supplementary material for: A scoping review of interventions to prevent and treat adverse events during treatment of rifampin-susceptible tuberculosis
Source: PLoS One. 2025 Dec 26;20(12):e0339354. doi: 10.1371/journal.pone.0339354 (PMC12742745; doi:10.1371/journal.pone.0339354)
Supplement: S8 Table — (DOCX) [file pone.0339354.s008.docx]

S8 Table. Summary of entries in clinical trials registries that include an objective to improve safety and tolerability of rifampin-susceptible tuberculosis treatment and whose results do not appear to have been published

| Title | Clinical Trials Number | Year of study proposal registration | Study type | Intervention evaluated | Sample size per arm or cohort size |
| --- | --- | --- | --- | --- | --- |
| Pharmacogenetics-guided Isoniazid Dosing in TB-HIV (PHINX) | NCT05124678 | 2021 | Prospective cohort | NAT2 fast and intermediate acetylators given10 mg/kg INH while slow acetylators receive 5 mg/kg | NR |
| A Pharmacogenetic Study for Isoniazid According to NAT2 Polymorphism Status | NCT03665402 | 2018 | Prospective cohort | NAT2 fast and intermediate acetylators given 300 mg INH while slow acetylators receive increased dose based on pharmacokinetics | NR |
| A double-blind, multicentre, parallel group, randomised, controlled trial to evaluate the possible benefit of isoniazid dose adjustment according to the genotype for NAT2 (arylamine N-acetyltransferase type 2) in patients with pulmonary tuberculosis | EUCTR2007-000224-41-BG | 2008 | Randomized trial | INH dose adjustment by NAT phenotype | NR |
| Isoniazid Dose Adjustment According to NAT2 Genotype (IDANAT2) | NCT00571753 | 2007 | Randomized trial | Isoniazid dose adapted according to NAT2 status i.e. appr. 2.5 mg/kg, 5 mg/kg and 7.5 mg/kg for slow, intermediate and rapid acetylators, respectively |  |
| N - acetyl transferase 2 encoding gene polymorphism in patients with tuberculosis application of accurate treatment | ChiCTR2300078685 | 2023 | Randomized trial | Fast acetylator - 1.5 times the standard dose of isoniazid.  Intermediate acetylator - standard dose of isoniazid. Slow acetylator - isoniazid dose adjusted using pharmacokinetic monitoring with target concentration of 3-6 mg/L 2 hours after the dose | 120 |
| Evaluation of the effects of vitamin C on reducing anti-tuberculous hepatotoxicity | IRCT20170904036060N | 2017 | Randomized trial | Group 1: vitamin C 500 mg/day for two months. Group 2: vitamin C 1000 mg/day for two months. Group 3 (Control group): placebo tablet daily for 2 months | 90 |
| Gene-guided N-acetyl Cysteine for prophylaxis of Anti-tuberculous Drug-induced Hepatitis | NCT06484530 | 2024 | Randomized trial | NAT2 slow acetylators randomized to NAC 600 mg twice daily for 8 weeks or no NAC | NR |
| Investigation of the Effect of N Acetylcysteine Against Anti-Tuberculosis Drugs Induced Liver Toxicity | NCT00564642 | 2007 | Retrospective cohort | N Acetylcysteine 1200 mg BD (BID?) for 2 weeks | NR |

NR – Not reported

NAT2 – N-acetyl transferase 2 enzyme
