# Supplementary material for: A scoping review of interventions to prevent and treat adverse events during treatment of rifampin-susceptible tuberculosis
Source: PLoS One. 2025 Dec 26;20(12):e0339354. doi: 10.1371/journal.pone.0339354 (PMC12742745; doi:10.1371/journal.pone.0339354)
Supplement: Attachment — Submitted filename: Burman_response letter_11 10.pdf [file pone.0339354.s010.pdf]

PONE-D-25-52218
A scoping review of interventions to prevent and treat adverse events during treatment of rifampin-susceptible tuberculosis
PLOS ONE

Dear Dr. Subbian,

We appreciate the careful review of our manuscript at PLOS ONE and the comments and suggestions from the peer reviewers. In the rebuttal letter below, our responses to requests and comments are indented and italicized, and changes in the wording of the manuscript are highlighted.

Regards, William J. Burman MD

Journal Requirements:
When submitting your revision, we need you to address these additional requirements.
1. Please ensure that your manuscript meets PLOS ONE's style requirements, including those for file naming. The PLOS ONE style templates can be found at https://journals.plos.org/plosone/s/file?id=wjVg/PLOSOne_formatting_sample_main_body.pdf and https://journals.plos.org/plosone/s/file?id=ba62/PLOSOne_formatting_sample_title_authors_affiliations.pdf

> *Response: We followed PLOS ONE's style requirements.*

2. Please provide a complete Data Availability Statement in the submission form, ensuring you include all necessary access information or a reason for why you are unable to make your data freely accessible. If your research concerns only data provided within your submission, please write "All data are in the manuscript and/or supporting information files" as your Data Availability Statement.

> *Response: We added the suggested Data Availability Statement to the manuscript.*

3. Please remove all personal information, ensure that the data shared are in accordance with participant consent, and re-upload a fully anonymized data set.
Note: spreadsheet columns with personal information must be removed and not hidden as all hidden columns will appear in the published file.
Additional guidance on preparing raw data for publication can be found in our Data Policy (https://journals.plos.org/plosone/s/data-availability#loc-human-research-participant-data-and-other-sensitive-data) and in the following article: http://www.bmj.com/content/340/bmj.c181.long.

> *Response: Not applicable.*

4. If the reviewer comments include a recommendation to cite specific previously published works, please review and evaluate these publications to determine whether they are relevant and should be cited. There is no requirement to cite these works unless the editor has indicated otherwise.

Response: Not applicable.

Additional Editor Comments:

REPORT from REVIEWER #3:

This is an interesting paper which addresses one of the series of questions raised by Dr Burman and his colleagues in their recent efforts to focus more attention on issues of safety and tolerability of current regimens for DS TB. This particular manuscript is a review of published trials and studies assessing interventions to mitigate some of the well known safety/tolerability challenges. As such, I think it represents a valuable contribution to the literature on this issue, and I support acceptance by PLOS ONE, following possible modest revision for the reasons noted below.
That said, I have a number of comments, questions and suggestions for the authors to consider in finalizing their submission.

ABSTRACT:
1. The Conclusions should arise from the Results reported. Several statements in the Conclusions paragraph are not supported by data presented in the Findings statements. One or the other should be revised.

Response: We agree and made the following changes in the Conclusions of the abstract: " There has been considerable research on improving the safety and tolerability of tuberculosis treatment, but its impact is limited by under-powered studies, the lack of inclusion of key subgroups, and important gaps in the research portfolio (uncertainties about the safety of pyrazinamide and the efficacy of hepatoprotectants, lack of research on ways to manage and prevent treatment-related nausea). It is concerning that the research pipeline for interventions to improve safety and tolerability appears to be quite limited Our review has identified promising interventions that may make treatment better tolerated, and hence, more effective".

INTRODUCTION:
Line 65: events

Response: Change made as suggested: "Despite the frequency of adverse events…"

METHODS:
Line 125: while it is not unexpected that a detailed analysis of study quality is not within the purview of a scoping review, the fact that sample sizes are often quite small raises concern about study quality. Is it

worthwhile to pursue findings of inadequately powered studies?

Response: We agree that clinical trials having < 50 patients/arm raise concerns about study quality, but we don't think there is a general answer to the question of whether the results of underpowered studies warrant further study. Each study needs to be reviewed in detail: participant selection, fidelity to the plan for intervention, endpoint definition, quality of follow-up during the intervention, trends in key endpoints, and results of other studies. We did not make changes to the manuscript regarding this comment.

RESULTS:
Line 156, Table 2: Half of the trials and studies had sample sizes below 60-100 per arm. It would be interesting to know if the risk or odds ratios were of substantial magnitude, even if the p values were not significant (given the relatively low power).

Response: Supplemental Table S4 provides some of these details. In response to the reviewer's specific question about randomized trials having < 100, we provide the following Table. Of note, not all trials provided results using risk or odds ratios. We did not add this table to the manuscript.

Of note, in reviewing these trials, we found one error in sample size (Dange, et al; sample size of 62 per arm, rather than 145). This correction changed the interquartile range (from 30 to 120, rather than 30 to 131. These changes were made in the Table 2 and in Table S4.

Summary of results of randomized clinical trials having sample sizes < 100 participants/arm

First author	Title	Study category, per the framework in Table 1	Sample size per arm	Endpoint(s) related to safety / tolerability	Brief summary of the study outcome
Wardhan	A study of the oxidative stress and the role of antioxidants in ATT induced hepatotoxicity in tuberculosis patients	Treatment of hepatotoxicity	5	Levels of "oxidative stress", ALT, AST levels	The group who received Vitamin E plus vitamin C had lower indices of oxidative stress; no differences in clinical outcomes
Hsieh	Efficacy of acupressure to prevent adverse reactions to anti-tuberculosis drugs: randomized controlled trials	Other form of prevention (details in the Comment variable	16	Adverse events	Fewer adverse skin reactions in the group who received acuppressure using anatomic points thought to help adverse reactions vs. those who had sham acuppressure
Saigal	Safety of an ofloxacin-based antitubercular regimen for the treatment of tuberculosis in patients with underlying chronic liver disease: a preliminary report	Change in PZA use	16	DILI, adverse events	DILI more common with IRE than IZEOflox (26.6% vs. 0, p - 0.043)
Maddahi	The efficacy of Jujube syrup on the prevention of drug-induced hepatotoxicity in pulmonary tuberculosis patients: A pilot randomized double-blind placebo-controlled clinical trial	Prevention of hepatoxicity	17	Nausea, anorexia, quality of life	Fewer patient on Jujube syrup had DILI (3, 0); lower AST at week 2 in experimental group; lower frequency of cough; no differences in nausea or anorexia
Mahani	Antiemetic activities of Indonesian stingless bee propolis on emetic induced by anti-tuberculosis drugs	Prevention of GI symptoms	17	Nausea (no details on how it was assessed)	Greater decrease in nausea at week 24 in the two intervention arms (no statistical test provided)
Talebi	The Effectiveness of Silymarin in the Prevention of Anti-tuberculosis Drug-induced Hepatotoxicity: A Randomized Controlled Clinical Trial	Prevention of hepatoxicity	18	DILI	ALT and ALP levels in the experimental group declined, while they increased in the control group; DILI occurred in 0 and 2 patients, respectively
Sukumaran	A study to evaluate the hepatoprotective effect of N- acetylcysteine on anti tuberculosis drug induced hepatotoxicity and quality of life	Prevention of hepatoxicity	19	Liver tests, quality of life, adverse drug reactions, oxidative stress biomarkers	Between group analysis showed, significant reduction in ALT ($p < 0.05$) and AST ($p < 0.05$) in NAC group at 4 weeks

Yazdani	The effects of livercare tablet [Combination of milk thistle, dandelion, barberry, tumeric (Curcumin), and artichoke] in prevention of anti-tuberculosis drugs-induced hepatotoxicity: A randomized controlled clinical trial	Prevention of hepatoxicity	22	DILI	6/22 patients (27%) in the control group developed DILI compared with 1/22 (5%) in the multiherbal group ($p = 0.039$)
Safe	Safety and efficacy of N-acetylcysteine in hospitalized patients with HIV-associated tuberculosis: An open-label, randomized, phase II trial (RIPENACTB Study)	Prevention of hepatoxicity	25	Tolerability, adverse events	Increased nausea and hepatotoxicity in NAC patients ($p > 0.05$)
Marjani	Evaluation of Silymarin for management of anti-tuberculosis drug induced liver injury: A randomized clinical trial	Treatment of hepatotoxicity	28	Severity of DILI, mortality, hospital stay, severity of liver injury, clinical complications of DILI	No differences in any of the endpoints
Emrani	Ginger for Prevention of Antituberculosis-induced Gastrointestinal Adverse Reactions Including Hepatotoxicity: A Randomized Pilot Clinical Trial	Prevention of GI symptoms	30	DILI	Nausea was less common among participants on ginger (70% vs. 90%, $p = 0.05$). Trend toward less vomiting and DILI in participants on ginger (17% vs. 37% for DILI; 27% vs. 53% for vomiting)
Kolomoiets	The effect of the preparation Wobenzym on the antioxidant protection indices and on the functional-morphological properties of the erythrocytes in a toxic lesion of the liver	Treatment of hepatotoxicity	30	ALT, AST, bilirubin	More rapid normalization of liver enzymes but no change in clinical events
Chen	Clinical study of deoxyribonucleotidum for adjuvant treatment of pulmonary tuberculosis with hepatic lesion	Treatment of hepatotoxicity	40		Patients were enrolled if ALT was > 40 during TB treatment. Those who received deoxyribonucleotidum together with HRZ had improved ALT, AST, and ALP at 4 weeks, compared to HRZ alone ($p <0.05$).
Mai	A randomised double blind placebo controlled phase 2 trial of adjunctive aspirin for tuberculous meningitis in HIV-uninfected adults	Corticosteroid or other immunomodulator	40	GI bleeding or cerebral bleeding	No statistically significant difference in grade 3+ events, though a trend toward increased AEs in the low-dose aspirin arm

Misra	Standard versus sequential anti-tubercular treatment in patients with tuberculous meningitis: a randomized controlled trial	Prevention of hepatoxicity	40	Death, mortality, disability	Decreased DILI with graded, sequential initiation (20 vs. 42.5%), lower inpatient mortality and days hospitalized, decreased paradoxical worsening, no difference in other adverse events
Yeswanth	A prospective single-blinded study on the safety and efficacy of zinc supplementation in pulmonary tuberculosis	Other form of prevention (details in the Comment variable	40	Adverse events	There was a trend decreased "GI disturbances" among participants on zinc (p = 0.06), but a trend in the opposite direction for constipation
Hakimizad	The Effect of acetyl-L-carnitine, Alpha-lipoic Acid, and Coenzyme Q10 Combination in Preventing Anti-tuberculosis Drug-induced Hepatotoxicity: A Randomized, Double-blind, Placebo-controlled Clinical Trial	Prevention of hepatoxicity	44	DILI	Lower incidence of DILI in experimental arm (6.8% vs. 25.6%); most cases were mild
Hagiwara	Safety of pyrazinamide-including regimen in late elderly patients with pulmonary tuberculosis: A prospective randomized open-label study	Change in PZA use	45	DILI, death, elevation of AST/ALT	Higher rate of discontinuation due to DILI in the HRE group (15.6% vs. 9.1%), but this did not reach statistical significance; no difference in liver injury > 2.5 x ULN; higher death rate with PZA (10 vs. 3, p = 0.032)
Du	Efficacy and safety of bicyclol for treating patients with antituberculosis drug-induced liver injury	Treatment of hepatotoxicity	49	adverse events	ALT values normalized more rapidly in the low-dose and high-dose bicyclol arms than in the control arm; no difference in clinical adverse events; no mention of ability to re-introduce TB drugs
Kamal	Prospective, Open Labelled, Randomised, Parallel Group Study To Evaluate The Efficacy And Safety Of Metformin Add-On Therapy To Standard ATT In Newly Diagnosed Pulmonary Tuberculosis Patients	Other form of prevention (details in the Comment variable	50	Adverse events (not classified using a standard system)	No differences in clinical or laboratory adverse events
Meintjes	Randomized placebo-controlled trial of prednisone for paradoxical tuberculosis-associated immune reconstitution inflammatory syndrome	Corticosteroid or other immunomodulator	55	IRIS symptoms, adverse events related to prednisone	Decrease in the primary endpoint; improvement in IRIS symptoms and quality of life, greater improvement in chest radiograph

Misra	Role of aspirin in tuberculous meningitis: a randomized open label placebo controlled trial	Corticosteroid or other immunomodulator	59	Death, functional status, pain abdomen, gastrointestinal hemorrhage, allergy, ecchymosis, purpura, jaundice and liver function abnormalities	Decreased stroke by MRI (43.3%) in the placebo and 24.2%) in the aspirin group; decreased mortality, improved functional outcome, no difference in side effects
Bunkar	Add-on prednisolone in the management of cervical lymph node tuberculosis	Corticosteroid or other immunomodulator	60	Symptoms at 2 and 6 months, complications during therapy, adverse events (nausea, rash, joint pain, etc.)	Improvements in symptom relief at 2 and 6 months, decreased complications (abscess, sinus new adenopathy). No difference in side effects.
Chuchottaworn	Adverse drug reactions and outcome of short course anti-tuberculosis drugs between single daily dose and split drug dose (BID) in pulmonary tuberculosis	Change in dosing frequency	61	Adverse drug reactions (severity not defined)	Participants on split dose treatment had a lower frequency of adverse drug reactions (52.5% vs. 62.3%)and somewhat higher frequency of cure (84.6% vs. 72.9%), but these differences did not reach statistical significance.
Dange	Efficacy of stimuliv, an indigenous compound formulation, against hepatotoxicity of antitubercular drugs--a double blind study	Prevention of hepatotoxicity	62	DILI	Decreased frequency of symptomatic and subclinical hepatitis (as determined by liver tests): 8% vs. 27%; all patients recovered and were able to resume TB treatment (HRZ)
Cross	Rosuvastatin adjunctive therapy for rifampicin-susceptible pulmonary tuberculosis: a phase 2b, randomised, open-label, multicentre trial	Prevention of hepatotoxicity	68	Grade 3+ AEs	No difference in AEs, including DILI
Adewole	Atorvastatin improve sputum conversion and chest x-ray severity score	Prevention of hepatotoxicity	75	Adverse events, QT changes	Increased muscle pain in atorvastatin group, increased neuropathy symptoms in controls; no difference in DILI
Azuma	NAT2 genotype guided regimen reduces isoniazid-induced liver injury and early treatment failure in the 6-month four-drug standard treatment of tuberculosis:	Change in INH use/dose	86	Other adverse events during the first 2	Decrease in combined unfavorable outcomes at 2 months (17% vs. 48%), decreases in INH-DILI and treatment failure. Specifically, the

	a randomized controlled trial for pharmacogenetics-based therapy.			months of treatment	decrease in INH-related DILI was among slow-acetylators
Sharma	Six months versus nine months anti-tuberculous therapy for female genital tuberculosis: a randomized controlled trial	Other change in the regimen (details in the Comment variable)	88	Adverse events	No trend regarding adverse events: 6 months - 33%; 9 months - 31%
Makharia	Intermittent Directly Observed Therapy for Abdominal Tuberculosis: A Multicenter Randomized Controlled Trial Comparing 6 Months Versus 9 Months of Therapy	Other change in the regimen (details in the Comment variable)	99	Side effects - not otherwise specified	No difference is side effects (21.3% with 6 months, 18.2% with 9 months)

Line 183: Sentence beginning with "However…" lacks its independent clause.

> *Response: This sentence was corrected to "However, three cohort studies did not find increased hepatoxicity…"*

Line 189, Table 3: The apparent finding of opposite effects from PZA in different studies is striking. There is substantial literature on the toxicity of PZA, and on the relation of dose to hepatotoxicity.

> *Response: We agree that the marked differences in reported outcomes of key adverse events (hepatotoxicity, nausea) in clinical trials and cohort studies of PZA are puzzling. As noted in the Discussion, this question needs to be resolved in randomized trials including study participants who are representative of the global tuberculosis pandemic and with appropriate statistical power.*

Line 218: the authors are certainly aware that intermittent therapy has been linked to lower efficacy. One of the authors co-authored an IPDMA demonstrating this some 15 years ago.

> *Response: We included references to the meta-analyses that showed decreased efficacy with intermittent therapy and noted this result: "Tuberculosis treatment guidelines now recommend daily therapy, based on meta-analyses showing better efficacy for daily vs. intermittent therapy." We did not make changes to the manuscript based on this comment.*

Line 220ff: interesting approaches such as those cited here (split dosing, alternate-day dosing) were suggested long ago, but have not yet been pursued. Such approaches may be of particular interest with drugs linked to toxicity, such as PZA.

> *Response: We agree that split dose and alternate day dosing deserve additional study as a way to manage some adverse events. We did not make changes to the manuscript based on this comment.*

Line 292: Table 5: the observation that several studies have found incremental reintroduction safer than simultaneous full dose reintroduction is a very useful observation, thank you

> *Response: We agree and did not make changes to the manuscript based on this comment.*

DISCUSSION:
Line 352: while the MRC trials that first demonstrated the treatment shortening capacity of PZA, those trials were followed by several other larger trials (BTA2, USPHS-S21) which involved hundreds of patients. PZA was not included in recommended regimens prior to those trials.

Response: We appreciate the reminder about the larger trials, though our review of those trials did not suggest a resolution to the uncertainty about the safety and tolerability of PZA. These trials were not included in the manuscript as such because their results were included in the two meta-analyses that are included in the scoping review. We removed the phrase "small trials", and the revised sentence reads as follows: "…the treatment-shortening trials that led to its routine inclusion in treatment regimens did not include patients with extrapulmonary or paucibacillary tuberculosis."

Line 367: the reservations about NAT-2 guided therapy seem unduly negative. Especially in areas were slow acetylation is common, adaptation of the dose of INH might be helpful. Studies are underway involving real-time testing and dose-adjustment.

Response: We removed the word "substantial" from the summary of limitations so that it now reads: "However, the limitations of this approach include: the frequency of primary isoniazid resistance in some parts of the world, the programmatic complexity of customized dosing, and the inability to use this approach in settings that use combined formulations".

Line 394: unfortunately most of the success of cancer chemotherapy has relied upon drugs that are primarily metabolized by the cytochrome p450 system, esp cyp3A4. Such agents are challenging to use in TB treatment due to inductive features of rifampin and rifapentine (and to a much lesser degree, rifabutin)

Response: We added a sentence providing some details about the possibility for using anti-emetic drugs used in cancer chemotherapy for patients on rifampin-containing tuberculosis treatment: "While some anti-emetic drugs used in chemotherapy have unacceptable drug-drug interactions with rifampin (e.g., the Neurokinin 1 receptor antagonists) (155), other potent anti-emetic drugs have acceptable interactions with rifampin (e.g., ondansetron, olanzapine) (156, 157)."

Line 400: while co-morbid conditions that may affect TB treatment outcomes (alcohol addiction, diabetes, tobacco smoking) have been much discussed, there is quite limited evidence of success in managing these issues while on TB therapy. Coordinated management is complicated and demands additional resources. If the authors have suggestions this would be helpful.

Response: We agree that management of concomitant medical conditions during tuberculosis treatment can be difficult because of drug-drug interactions, the lack of resources for such management in tuberculosis treatment clinics, and the complexity of administering some forms of management (e.g., injectable long-acting naltrexone). We hope that our scoping review will spark interest in investigators who have expertise in management of chronic medical conditions such as diabetes and substance use disorders. We did not change the manuscript in response to this comment.

Line 418 and Line 202: higher doses of drugs (with rifamycins, with PZA, or with children) illustrate the challenge that "there is no free lunch," with larger doses usually implying greater risk for adverse events.

Response: We agree. We did not change the manuscript in response to this comment.

CONCLUSIONS: These are nicely stated. I particularly appreciated the "both/and" suggestion. I suggest that either the authors consider enlarging on this concept, or that an accompanying editorial address the issues in such an approach. Relevant issues include ways to improve safety of the existing drugs (monitoring, assessing toxicity risk factors for specific drugs, varying the rhythms of drug administration, adding measures to mitigate or prevent adverse events, etc.), ways to improve safety of agents in development (eg. use of humanoid platforms for testing), the advisability of the implicit individualization of regimens (which may be needed to address population-specific vulnerabilities to particular AEs), improved surveillance of the nature and severity of adverse events to allow better assessment of risks and benefits, and mechanisms to support such research due to the substantial lack of commercial support.

Response: We agree that an editorial addressing the points raised by the reviewer would be very helpful. We revised the Conclusions paragraph, as well, "Treatment-related adverse events remain a major barrier to achieving cure at the individual level and tuberculosis control in the population. In the long term, safer and more effective drugs are needed, but our scoping review demonstrates that there are ample opportunities for studies using currently available drugs and other interventions. We urge the TB community to pursue further high-quality clinical research in this area using a both/and strategy – emphasizing the identification of safer, better-tolerated new drugs, and doing research on ways to improve the safety and tolerability of the treatment regimens we have now".

FIGURES AND TABLES: There is an inherent contradiction in this MS, in that there are multiple tables presenting multiple interventions whose studies are often very underpowered and of uncertain quality. As a result, I wonder if the tables could be made shorter, with the more detailed information in a supplement.

Response: We don't see an inherent contradiction in the tables in our manuscript. We used scoping review methodology to do a very broad overview of a neglected area of tuberculosis treatment – the prevention and management of treatment-related adverse events. The current tables are an attempt to balance brevity and the need to have at least some information from all studies that met the criteria for inclusion in the scoping review. In that Reviewer 3 asked shorter, simpler tables and Reviewer 1 asked for additional detail in those same tables, we think we did a reasonable job of finding the needed balance. Notably, Supplemental Table S4 is designed to provide additional detail to the reader who is interested in aspects of the papers, such sample size, the country in which the trial was done, and a short summary of the results. We did not change the manuscript in response to this comment.

[Note: HTML markup is below. Please do not edit.]

Reviewers' comments:

Reviewer's Responses to Questions
Comments to the Author

1. Is the manuscript technically sound, and do the data support the conclusions?

The manuscript must describe a technically sound piece of scientific research with data that supports the conclusions. Experiments must have been conducted rigorously, with appropriate controls, replication, and sample sizes. The conclusions must be drawn appropriately based on the data presented.
Reviewer #1: Yes
Reviewer #2: Yes

---

2. Has the statistical analysis been performed appropriately and rigorously?
Reviewer #1: N/A
Reviewer #2: N/A

---

3. Have the authors made all data underlying the findings in their manuscript fully available?

The PLOS Data policy requires authors to make all data underlying the findings described in their manuscript fully available without restriction, with rare exception (please refer to the Data Availability Statement in the manuscript PDF file). The data should be provided as part of the manuscript or its supporting information, or deposited to a public repository. For example, in addition to summary statistics, the data points behind means, medians and variance measures should be available. If there are restrictions on publicly sharing data—e.g. participant privacy or use of data from a third party—those must be specified.
Reviewer #1: No
Reviewer #2: Yes

---

4. Is the manuscript presented in an intelligible fashion and written in standard English?

PLOS ONE does not copyedit accepted manuscripts, so the language in submitted articles must be clear, correct, and unambiguous. Any typographical or grammatical errors should be corrected at revision, so please note any specific errors here.
Reviewer #1: No
Reviewer #2: Yes

---

5. Review Comments to the Author

Please use the space provided to explain your answers to the questions above. You may also include additional comments for the author, including concerns about dual publication, research ethics, or publication ethics. (Please upload your review as an attachment if it exceeds 20,000 characters)

Reviewer #1: The manuscript "A scoping review of interventions to prevent and treat adverse events during treatment of rifampin-susceptible tuberculosis" is interesting but requires modification to enhance the presentation and add interest to the readers.

1. Table 1 – Is "Other means of preventing adverse events" placed correctly? Is it referring to • Nausea/vomiting • Hepatotoxicity • Immunomodulatory agents - Please rephrase the section for clarity

> *Response: We appreciate this comment and changed the sub-title to "Other interventions to prevent specific adverse events" and indented that sub-title to clarify that it is under the section on "Prevention of adverse events". The upper portion of the revised section of Table 1 is below.*

Prevention of adverse events
Tuberculosis treatment regimen changes
• Pyrazinamide – dose, duration
• Isoniazid – dose, alternative drugs
• Rifamycin – choice, dose
• Ethambutol – duration, alternative drugs
• Dosing frequency – split dose, intermittent dosing
• Duration of therapy
Other interventions to prevent specific adverse events
• Nausea/vomiting
• Hepatotoxicity
• Immunomodulatory agents

2. In the results, under the section "Changes in the TB treatment regimen ", please try to include subsections as classified in Table 2 and 3

Response: Table 2 presents an overview of the study methodologies used and sample sizes. Tables 3-5 include notations about the study methodology used, but these tables are organized by topic (e.g., "dose of PZA"), rather than methodology. We did not make changes to the manuscript based on this comment.

3. Table 2 represents the classification of studies into 4 types. Table 3 has included details drug wise excluding unconventional drugs and limited to PZA, INH and FQ. Table 3 can be elaborated across the key findings – Expand the same information to include additional details like sample size, country, year and type of study in addition to findings.

Response: As noted above in one of the responses to Reviewer #3, Table 3-5 are a balance of the need for brevity and the need to include some information from each of the studies that met the criteria for inclusion in our scoping review. The details listed above are included in Supplementary Table S4 for the readers who is interested in more detailed information. We did not make changes to the manuscript based on this comment.

4. Please try to segregate information under appropriate tiles for the section "Prevention of adverse events by interventions other than changes in the treatment regimen"

Response: For clarity, we changed the title for Table 4 to be: "Summary of studies of interventions to prevent specific adverse events, other than changes in the treatment regimen". We think the first row of this table has the appropriate tiles for the material presented.

5. Add additional information to table 4 as well – like table 3

Response: See the response above to comment 3. We did not make changes to the manuscript based on this comment.

6. Discussion is elaborate need to focus on key messages and how to minimize adverse events. The author should elaborate more on the areas which require more intervention to manage adverse events.

Response: We used the Discussion section to highlight priority areas for further research: trials to clarity the safety and tolerability of pyrazinamide, trials to improve the prevention and treatment of nausea/vomiting, a trial of treatment initiation by dose escalation. We did not make changes to the manuscript in response to this comment.

7. Conclusion could add some summary of key findings and elaborate on the interventions or methodologies recommended way forward

Response: We agree and made changes to the Conclusion section to provide specific priority topics that deserve more research (see response to the first comment by Reviewer #3).

8. It would be a good idea to add a graphical summary or a diagrammatic representation of the content by flow or block diagram differentiating intervention strategies targeting the bacterial and/or host mechanism.

> *Response: Table 1 provides the theoretical framework that was used to organize our review. The Reviewer's suggested framework of strategies targeting the bacteria or the host does not seem relevant to this topic; we consider all of the interventions reviewed in the paper to be based on trying to change something about the host or the treatment regimen.*

Reviewer #2: In this review, the authors have reviewed the existing literature on the management of adverse events associated with anti-tuberculosis drug treatment among rifampicin-sensitive tuberculosis patients, with the objective of identifying interventions (either to prevent or manage those adverse events, either by changing the treatment regimen or by other means) and the ongoing research in this area. Indeed, it is a topic of importance and likely to attract interest, particularly from high TB burden countries. However, this scoping review has a few notable limitations in addition to those listed in the article.

There are several adverse events associated with anti-TB treatment, and they may vary among patients (including in severity); thus, interventions of any kind are unlikely to address all or most of the adverse events. Therefore, most of the studies involve targeted interventions.

> *Response: We agree that the interventions we reviewed are targeted and are are unlikely to address all adverse events. As above, we changed the title of Table 4 to clarity that these interventions are for specific adverse events.*

Although the authors have summarized several interventions from various studies that evaluated the management of adverse events, it is not clear to what extent these studies achieved successful management of such adverse events.

> *Response: Our view of the scoping review methodology is that its purpose is identify questions in need of further study, not to provide the answer to those questions. Tables 3-5 provide our summary of the results of individual studies, and Supplementary Table S4 provides more detail for the interested reader.*

It would have been interesting for further research if the authors had included key findings from the studies (e.g., percentage reduction in nausea or hepatotoxicity among study participants) for particular types of adverse events (Table 4; e.g., gastrointestinal adverse effects, hepatotoxicity, etc.) rather than simple statements. Otherwise, the table is merely a list of existing literature.

> *Response: We included more details about the results of individual studies in Supplementary Table S4. The reason for not including these details in Tables 3-5 were to maintain brevity and clarity. The key finding regarding the management of nausea/vomiting during tuberculosis*

treatment is that there is virtually no data on what is the most common treatment-related adverse event (two underpowered trials that used non-standard products). Rather than focusing on the specific outcomes of these underpowered studies, our scoping review aims to point out promising interventions and motivate investigators to do more to improve the safety and tolerability of tuberculosis treatment.

Overall, based on the set of questions framed by the authors, this scoping review provides a comprehensive compilation of the literature.
